# First Insight into the Modulation of Noncanonical NF-κB Signaling Components by Poxviruses in Established Immune-Derived Cell Lines: An In Vitro Model of Ectromelia Virus Infection

**DOI:** 10.3390/pathogens9100814

**Published:** 2020-10-04

**Authors:** Justyna Struzik, Lidia Szulc-Dąbrowska, Matylda B. Mielcarska, Magdalena Bossowska-Nowicka, Michał Koper, Małgorzata Gieryńska

**Affiliations:** 1 Division of Immunology, Department of Preclinical Sciences, Institute of Veterinary Medicine, Warsaw University of Life Sciences-SGGW, Ciszewskiego 8, 02-786 Warsaw, Poland; lidia_szulc@sggw.edu.pl (L.S.-D.); matylda_mielcarska@sggw.edu.pl (M.B.M.); magdalena_bossowska@sggw.edu.pl (M.B.-N.); malgorzata_gierynska@sggw.edu.pl (M.G.); 2 Institute of Genetics and Biotechnology, Faculty of Biology, University of Warsaw, A. Pawińskiego 5A, 02-106 Warsaw, Poland; mkoper@ibb.waw.pl

**Keywords:** dendritic cells, macrophages, antiviral immunity, noncanonical NF-κB signaling, ectromelia virus

## Abstract

Dendritic cells (DCs) and macrophages are the first line of antiviral immunity. Viral pathogens exploit these cell populations for their efficient replication and dissemination via the modulation of intracellular signaling pathways. Disruption of the noncanonical nuclear factor κ-light-chain-enhancer of activated B cells (NF-κB) signaling has frequently been observed in lymphoid cells upon infection with oncogenic viruses. However, several nononcogenic viruses have been shown to manipulate the noncanonical NF-κB signaling in different cell types. This study demonstrates the modulating effect of ectromelia virus (ECTV) on the components of the noncanonical NF-κB signaling pathway in established murine cell lines: JAWS II DCs and RAW 264.7 macrophages. ECTV affected the activation of TRAF2, cIAP1, RelB, and p100 upon cell treatment with both canonical and noncanonical NF-κB stimuli and thus impeded DNA binding by RelB and p52. ECTV also inhibited the expression of numerous genes related to the noncanonical NF-κB pathway and RelB-dependent gene expression in the cells treated with canonical and noncanonical NF-κB activators. Thus, our data strongly suggest that ECTV influenced the noncanonical NF-κB signaling components in the in vitro models. These findings provide new insights into the noncanonical NF-κB signaling components and their manipulation by poxviruses in vitro.

## 1. Introduction

Transcription factor nuclear factor κ-light-chain-enhancer of activated B cells (NF-κB) regulates genes that are involved in the various mechanisms of the innate antiviral immune response [1]. The canonical NF-κB signaling pathway can be induced by numerous stimuli: (1) by the pathogen-associated molecular patterns (PAMPs) activating Toll-like receptors (TLRs) and other pattern recognition receptors (PRRs); (2) by the proinflammatory cytokines, such as tumor necrosis factor-α (TNF-α) and interleukin-1β (IL-1β) [2,3]; (3) by interferon-γ (IFN-γ); and (4) by phorbol 12-myristate 13-acetate (PMA) [4]. After the activation of the canonical NF-κB signaling pathway, transforming growth factor β-activated kinase 1 (TAK1) triggers the phosphorylation of the inhibitor of κBα (IκBα) kinase (IKK). Activated IKK, in turn, leads to the phosphorylation and proteasomal degradation of IκBα. Degradation of IκBα results in the rapid translocation of RelA (p65)/p50 and c-Rel/p50 heterodimers from the cytoplasm to the nucleus to regulate the transcription of the genes involved in innate immunity and inflammation [3].

The noncanonical NF-κB signaling pathway can be activated by ligands belonging to the TNF superfamily [5]. However, canonical NF-κB activators, such as lipopolysaccharide (LPS), TNF-α, IFN-γ, and PMA may also induce the noncanonical NF-κB signaling pathway [6]. In addition, bacterial lipopeptide and its synthetic counterpart, Pam3CSK4, may activate RelB, which is involved in the noncanonical NF-κB signaling [7]. The activation of the noncanonical NF-κB signaling pathway is based on the stabilization of its central component, NF-κB-inducing kinase (NIK). Under basal conditions, NIK interacts with TNF receptor-associated factor 3 (TRAF3) and is continuously degraded by E3 ubiquitin ligase complex composed of cellular inhibitor of apoptosis protein 1/2 (cIAP1/2)–TRAF2–TRAF3. During the activation of the noncanonical NF-κB signaling pathway, NIK is stabilized as a result of cIAP1/2- and TRAF2-induced TRAF3 degradation. NIK, in turn, induces the phosphorylation of IKKα, leading to the proteolytic degradation of p100 and the release of RelB/p52 dimer, which then translocates to the nucleus to control the transcription of the target genes [3,8,9,10]. Thus, the noncanonical NF-κB signaling pathway orchestrates the development of lymphoid organs, humoral immune response, function of T cells and dendritic cells (DCs), progression of inflammatory diseases, and antiviral innate immunity [5]. In recent years, strong evidence has emerged to support the role of noncanonical NF-κB activation in antiviral immunity. Interestingly, the noncanonical NF-κB signaling controls histone modification, thereby preventing the binding of RelA to the *Ifnb* promoter. As a result, type I IFN (IFN-I) activation and consequent antiviral innate response is attenuated [11].

Both innate and adaptive antiviral immune responses can be studied using antigen-presenting cells (APCs), such as DCs and macrophages, which link innate and adaptive immunity [12]. Importantly, the noncanonical NF-κB signaling is involved in the functioning of these cells [7,13,14,15,16,17,18]. DCs and macrophages play a key role in the antiviral immune response. However, at the same time, they serve as reservoirs of the virus [19,20]. Viral pathogens, in turn, modulate host signaling pathways to inhibit inflammatory response or apoptosis, which are regulated by the NF-κB signaling pathway. Modulation of the noncanonical NF-κB activation pathway is attributed to oncogenic viruses, whose products may interact with the components of both canonical and noncanonical NF-κB signaling pathways [1,10]. Some of the nononcogenic RNA viruses, including influenza A virus (FLUAV), human respiratory syncytial virus (HRSV), human enterovirus 71 (EVA71), bovine foamy virus (BFV), rotavirus, rabies virus (RABV), Sindbis virus (SINV), and DNA viruses including human herpesvirus 3 (HHV-3) and Orf virus (ORFV), influence the noncanonical activation of NF-κB [21].

**Figure 1:**
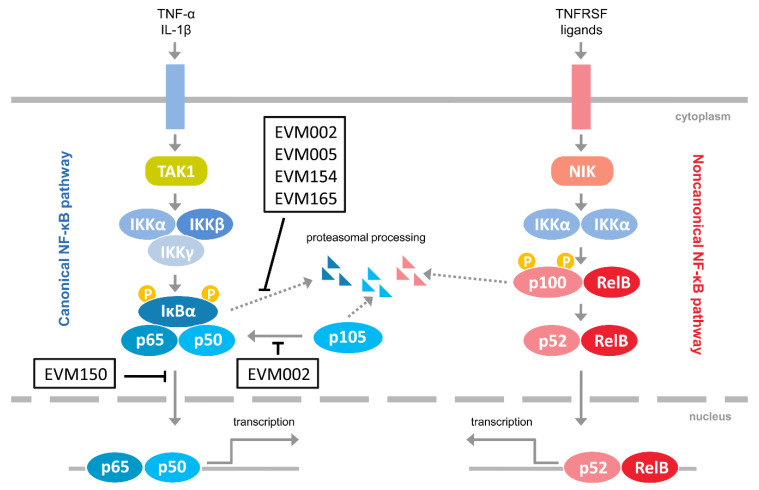
Inhibitors of NF-κB encoded by ectromelia virus (ECTV). The figure represents ECTV-encoded proteins that have been shown to interfere with NF-κB signaling [23,24,25,26]. Pointed arrows indicate activation; blunted arrows indicate inhibition. EVM002, EVM005, EVM154, EVM165, Ank/F-box proteins; EVM150, Kelch repeat, and BTB domain-containing protein 1; IL-1β, interleukin-1β; IKKα, inhibitor κB kinase α subunit; IKKβ, inhibitor κB kinase β subunit; IKKγ, inhibitor κB kinase γ subunit; NIK, NF-κB-inducing kinase; TAK1, transforming growth factor β-activated kinase 1; TNFRSF, tumor necrosis factor receptor superfamily; TNF-α, tumor necrosis factor α.

Considering the emerging role of the noncanonical NF-κB activation in antiviral innate immunity, as well as the fact that it regulates the canonical NF-κB signaling [11], we investigated how ectromelia virus (ECTV) influences the activation of the noncanonical NF-κB signaling components. ECTV is a pathogen of mice, belonging to the *Poxviridae* family and *Orthopoxvirus* genus. It is closely related to variola virus (VARV), a causative agent of smallpox and vaccinia virus (VACV), which was used as a vaccine against smallpox. Inhibition of NF-κB signaling by the members of the *Poxviridae* family has been studied extensively [22]. Figure 1 summarizes the modulation of NF-κB signaling by ECTV-encoded proteins [23,24,25,26]. Due to its similarities in genetic background and disease presentation with smallpox, mousepox (smallpox of mice) is recognized as an excellent model to study smallpox infection in humans, zoonotic monkeypox, as well as generalized viral infections. Importantly, a mousepox model is also used for testing medical countermeasures against VARV and other orthopoxviruses [27,28].

Our previous reports demonstrate that ECTV affects the canonical NF-κB signaling pathway in DCs and macrophages [28,29]. Other studies have revealed the role of NF-κB in resistance to ECTV infection in B6 mice. In inflammatory monocytes, ECTV infection activates NF-κB, which induces the expression of IFN-β, thus conferring antiviral immunity [30]. In this study, for the first time, we focused on the noncanonical NF-κB signaling components in established immune-derived cell lines that are permissive for ECTV infection: RAW 264.7 macrophages and JAWS II DCs [28,29].

Our results showed that ECTV modulates the cellular content of TRAF2 and cIAP1, counteracts the activation of p100 and RelB, inhibits the nuclear translocation of RelB, and affects the p100/p52 ratio in JAWS II DC and RAW 264.7 macrophage cell lines. Importantly, ECTV downregulated numerous RelB-dependent genes and inhibited the expression of selected genes encoding the components of the canonical and noncanonical NF-κB signaling pathway. Taken together, our data suggest that ECTV actively influences the noncanonical NF-κB signaling components, thus uncovering the new targets of viral manipulation.

## 2. Materials and Methods

### 2.1. Virus

In this study, the Moscow strain of ECTV (ECTV-MOS; ATCC, VR-1374; ATCC, Manassas, VA, USA) was used. The virus was propagated and titrated by plaque-formation assay in Vero cell line (ATCC, CCL-81) (ATCC, Manassas, VA, USA), and the final titer was expressed as plaque-forming units per mL (PFU/mL). After this procedure, the virus was aliquoted and stored at −70 °C until use. 

### 2.2. Cell Culture, Infection, and Treatment

JAWS II DCs (ATCC, CRL-11904) (ATCC, Manassas, VA, USA) and RAW 264.7 macrophages (ATCC, TIB-71) (ATCC, Manassas, VA, USA) were cultured as previously described [29,31]. 

For our experiments, both cell lines were seeded at an appropriate density into 24-well plates containing round microscopic slides (2 × 10^5^ JAWS II cells or 3 × 10^5^ RAW 264.7 cells), 6-well plates (2 × 10^6^ JAWS II cells or 1.5 × 10^6^ RAW 264.7 cells), or 25 cm^2^ flasks (5 × 10^6^ RAW 264.7 cells), and maintained for 24 h before the experiment.

For immunofluorescence analysis, cells were left uninfected (mock) or were infected with UV-inactivated ECTV (uvi-ECTV) or live ECTV. The multiplicity of infection (m.o.i.) was 0.7 for JAWS II cells or 2 for RAW 264.7 cells. JAWS II cells were also treated with 100 ng/mL of PMA (Sigma-Aldrich, St. Louis, MO, USA) + 1 μM ionomycin (Io) (Sigma-Aldrich, St. Louis, MO, USA) for 12 h. RAW 264.7 macrophages were stimulated with either 100 ng/mL of PMA + 1 μM of Io for 12 h, or 1 μg/mL of LPS (*Escherichia coli* O111:B4) (Sigma-Aldrich, St. Louis, MO, USA) + recombinant mouse (rm) IFN-γ (2.5 ng/mL) (Sigma-Aldrich, St. Louis, MO, USA) for 4 and 18 h or LPS alone for 24 h. Following stimulation, the cells were fixed and analyzed for the nuclear translocation of RelB.

For Western blot analysis, cells were mock- and ECTV-infected at an m.o.i. of 1 (JAWS II) and 2 (RAW 264.7). For the detection of phospho-proteins (p-p100 and p-RelB), JAWS II cells were stimulated with 100 ng/mL of PMA + 1 μM of Io for 15 min or 1 μg/mL of LPS O111:B4 + 2.5 ng/mL of rmIFN-γ for 30 min before harvesting 18 h postinfection (hpi). RAW 264.7 cells were stimulated for 45 min with 1 μg/mL of Pam3CSK4 or 1 μg/mL of LPS O111:B4 + 2.5 ng/mL of rmIFN-γ and harvested at 18 hpi. For analyzing the content of noncanonical NF-κB signaling components, JAWS II cells were treated with 50 μg/mL of polyinosinic:polycytidylic acid (poly(I:C); InvivoGen, San Diego, CA, USA), or 1 μg/mL of LPS O111:B4 + 2.5 ng/mL of rmIFN-γ and harvested at 18 hpi. RAW 264.7 cells were treated with 50 μg/mL of poly (I:C), 1 μg/mL of Pam3CSK4 (InvivoGen, San Diego, CA, USA), or 1 μg/mL of LPS (*E. coli* O55:B5 or O111:B4) (Sigma-Aldrich, St. Louis, MO, USA), in addition to 2.5 ng/mL of rmIFN-γ and harvested at 4 and 18 hpi. 

For analyzing the content of nuclear and cytoplasmic proteins, RAW 264.7 cells were left uninfected or were infected with ECTV-MOS at an m.o.i. of 2. Cells were left unstimulated or were stimulated with 1 μg/mL of LPS O111:B4 alone for 18 h or with 2.5 ng/mL of rmIFN-γ + 1 μg/mL of LPS O111:B4 for 4 and 18 h and then harvested.

For the quantitative PCR analysis, RAW 264.7 cells were left uninfected or were infected with ECTV-MOS at an m.o.i. of 1. The cells were left unstimulated or were stimulated with either 50 μg/mL of poly(I:C) or 1 μg/mL of LPS O111:B4 + 2.5 ng/mL of rmIFN-γ and harvested at 18 hpi.

### 2.3. Immunofluorescence Staining Technique and Microscopic Analysis

Paraformaldehyde (PFA) (Sigma-Aldrich, St. Louis, MO, USA) solution (4% in phosphate-buffered saline (PBS) (Sigma-Aldrich, St. Louis, MO, USA) was used for cell fixation. Cells were then permeabilized with 0.5% Triton X-100 (Sigma-Aldrich, St. Louis, MO, USA) in PBS for 30 min and blocked with 3% bovine serum albumin (BSA) (Sigma-Aldrich, St. Louis, MO, USA) in 0.1% Triton X-100 in PBS for 1 h. For RelB detection, cells were incubated with anti-RelB rabbit monoclonal primary antibody (mAb) (EPR7076)—C-terminal (Abcam, Cambridge, MA, USA)—for 1 h. Slides were then washed with 0.1% Triton X-100 in PBS and incubated with goat anti-rabbit IgG-Rhodamine RedX (Jackson ImmunoResearch Laboratories, Inc., West Grove, PA, USA) secondary polyclonal antibody (pAb) in 3% BSA in 0.1% Triton X-100 in PBS for 1 h. Following incubation, ECTV antigens were detected by adding anti-ECTV-FITC pAbs for 1 h. DNA was visualized by Hoechst 33342 (Calbiochem, San Diego, CA, USA) staining (1 μg/mL for 10 min). Slides were mounted in ProLong Gold Antifade Reagent (Invitrogen/Life Technologies, Carlsbad, CA, USA). Images were captured using an Olympus BX60 fluorescence microscope (Olympus, Tokyo, Japan) equipped with a Color View III cooled CCD camera (Olympus, Tokyo, Japan). The obtained images were analyzed using Cell^F (Soft Imaging System, Olympus, Tokyo, Japan), ImageJ (NIH, Bethesda, Maryland, USA), CellSens Dimension (Olympus, Tokyo, Japan), and Adobe Photoshop CS2 (Adobe Systems Inc., San Jose, CA, USA) software. Cells with greater fluorescence intensity in the nucleus than that of cytoplasm were counted as RelB-positive. A total of 50 (JAWS II) or 100 (RAW 264.7) cells/condition and experiment were analyzed. The slides were obtained from three independent experiments.

### 2.4. Western Blot Analysis

For Western blot analysis, cells were harvested with RIPA buffer (Thermo Fisher Scientific, Waltham, MA, USA) supplemented with 1% protease inhibitor cocktail (Thermo Fisher Scientific, Waltham, MA, USA). Protein concentration in cell lysates was measured using a QuantiPro BCA Assay Kit (Sigma-Aldrich, St. Louis, MO, USA) and Epoch BioTek spectrophotometer (BioTek Instruments, Inc., Winooski, VT, USA). Equal amounts of proteins were loaded onto polyacrylamide Bis-Tris Plus gel (Thermo Fisher Scientific, Waltham, MA, USA). Electrophoresis was run with MES running buffer (Thermo Fisher Scientific, Waltham, MA, USA). The proteins were transferred onto polyvinylidene fluoride (PVDF) membrane (Bio-Rad, Hercules, CA, USA). The membrane was blocked using 5% BSA in Tris-buffered saline and Tween 20 (TBST) for 1 h at room temperature. Next, the membranes were incubated overnight at 4 °C with primary rabbit mAbs, including anti-glyceraldehyde 3-phosphate dehydrogenase (GAPDH; loading control) D16H11; anti-NIK (EPR18588); anti-p-RelB (Ser552) (D41B9) (Cell Signaling Technology, Danvers, MA, USA) and anti-RelB (EPR7076)—C-terminal (Abcam, Cambridge, MA, USA); and rabbit pAbs, including anti-c-IAP1, anti-TRAF2, anti-TRAF3, anti-NF-κB2 p100/p52 (Cell Signaling Technology, Danvers, MA, USA), and anti-p-NF-κB p100 (pSer872) (Sigma-Aldrich, St. Louis, MO, USA). After incubation with primary antibodies, the membranes were washed with TBST and incubated with secondary goat anti-rabbit-IgG pAbs conjugated with horseradish peroxidase (HRP) (Cell Signaling Technology, Danvers, MA, USA). Proteins were detected by chemiluminescence using Clarity Western ECL Substrate (Bio-Rad, Hercules, CA, USA). The film images were captured with transillumination using a 5MP (mega pixels) camera. The images were converted to grayscale (TIFF). Next, the images were converted to 8-bit using ImageJ (NIH) software for densitometric analysis. The signal intensities of the analyzed bands were measured and normalized against cytoplasmic/whole cell (GAPDH) or nuclear (PARP) loading controls using ImageJ software. The normalized signal intensities were then compared to the normalized intensity of the control band expressed as 100%.

### 2.5. Nuclear and Cytoplasmic Cell Fractionation and Detection of Cytoplasmic and Nuclear Proteins

To obtain nuclear and cytoplasmic fractions, NE-PER nuclear and cytoplasmic extraction reagents (Thermo Fisher Scientific, Waltham, MA, USA) were used. Protein concentrations in the nuclear and cytoplasmic fractions were measured using a QuantiPro BCA Assay Kit (Sigma-Aldrich, St. Louis, MO, USA) and Epoch BioTek spectrophotometer (BioTek Instruments, Inc., Winooski, VT, USA). RelB and p52 NF-κB were detected in both fractions by Western blot analysis using primary anti-RelB (Abcam, Cambridge, MA, USA) and anti-NF-κB2 p100/p52 antibodies (Cell Signaling Technology, Danvers, MA, USA) and secondary goat anti-rabbit-IgG-HRP pAbs (Cell Signaling Technology, Danvers, MA, USA). Anti-GAPDH rabbit mAbs (Cell Signaling Technology, Danvers, MA, USA) were used as a cytoplasmic loading control, and anti-poly(ADP ribose) polymerase (PARP) (46D11) rabbit mAbs (Cell Signaling Technology, Danvers, MA, USA) were used as a nuclear loading control.

### 2.6. DNA-Binding ELISA

For the analysis of DNA binding by RelB and p52 NF-κB subunits, extracts containing nuclear proteins were obtained by cell fractionation (as described in Section 2.5). The extracts were analyzed by a DNA-binding ELISA using TransAM^®^ NFκB Family kit (Active Motif, Carlsbad, CA, USA) according to the manufacturer’s protocol. The activity of RelB and p52 NF-κB was determined based on the optical density (OD) of the reaction mixture at 450 nm.

### 2.7. RNA Isolation

Total RNA was isolated using the High Pure RNA Isolation Kit (Roche Diagnostics GmbH, Mannheim, Germany). RNA concentration and purity were assessed using a Take-3 system on an Epoch BioTek spectrophotometer (BioTek Instruments, Inc., Winooski, VT, USA), and the data were analyzed in Gen5 software (BioTek Instruments, Inc., Winooski, VT, USA). RNA integrity number (RIN) was determined to evaluate the integrity and level of degradation of RNA using an Agilent 2100 Bioanalyzer and Agilent RNA 6000 Pico Kit (Agilent Technologies, Palo Alto, CA, USA). RIN values were equal to or higher than 9. 

### 2.8. Reverse Transcription and Quantitative PCR (RT-qPCR)

Total RNA (1 μg) was reverse-transcribed to cDNA using the Transcriptor First Strand cDNA Synthesis Kit (Roche Diagnostics GmbH, Mannheim, Germany). Selected genes engaged in the regulation of the NF-κB signaling pathway were analyzed using a RealTime ready Custom Panel (Roche Diagnostics GmbH, Mannheim, Germany), containing 88 target genes, three reference genes (*Rn18s*, *Gusb*, and *Gapdh*), and five control genes (three positive control genes (each targeting different portions of the same transcript: at the 3′ end, in the middle, and at the 5′ end) and two negative control genes, determining the presence of genomic DNA). Each assay includes primers and a Universal ProbeLibrary (UPL) Probe preplated in the wells of the plate. The total amount of 550 ng cDNA was mixed with LightCycler 480 Probes Master mix (Roche Diagnostics GmbH, Mannheim, Germany) and aliquoted into the 384-well plates. Amplification was performed using a LightCycler 480 instrument (Roche Diagnostics GmbH, Mannheim, Germany) at 95 °C for 10 s, 60 °C for 30 s, and 72 °C for 1 s during 45 cycles, following an initial preincubation at 95 °C for 10 min. The fold change in target mRNA was calculated using the 2^−ΔΔCt^ method following normalization to *Rn18s*. All RT-qPCR experiments were performed according to the Minimum Information for Publication of Quantitative Real-Time PCR Experiments (MIQE) guidelines [32].

### 2.9. Statistical Analysis

STATISTICA 6.0 (StatSoft Inc., Tulsa, OK, USA) software was used for statistical analysis of the obtained data. Results of at least two (densitometric analysis) or three independent experiments (fluorescence microscopy, DNA-binding ELISA, or RT-qPCR) are shown as the mean ± standard deviation (SD). Paired and unpaired *t*-tests were used in the evaluation of the significance of differences between the analyzed groups (*p* ≤ 0.05 (*) and *p* ≤ 0.01 (**) indicate statistical significance).

## 3. Results

### 3.1. ECTV Affects RelB and p52 Nuclear Translocation in RAW 264.7 Macrophages at an Early Stage of Viral Replication

RAW 264.7 cells are highly permissive to ECTV infection. After 4 h of cell infection with ECTV at MOI = 2, RAW 264.7 macrophages display the presence of virions entering the cells or viral replication sites called viral factories. Since ECTV induces apoptosis of RAW 264.7 cells, the cells were infected at an m.o.i. of 2 to prevent disruption of the cell membranes before 18 hpi [29].

Firstly, we analyzed the effect of ECTV on subcellular localization of RelB and p52 in RAW 264.7 cells at 4 hpi. RAW 264.7 cells are characterized by very low expression of CD40, a member of the TNF superfamily, which induces the noncanonical NF-κB signaling in macrophages [33,34]. Therefore, to induce the activation of the noncanonical NF-κB signaling components, we treated the cells with IFN-γ + LPS. It has been shown that nuclear translocation of RelB and, to a lesser extent, RelA, is induced by IFN-γ + LPS in RAW 264.7 macrophages [35].

**Figure 2:**
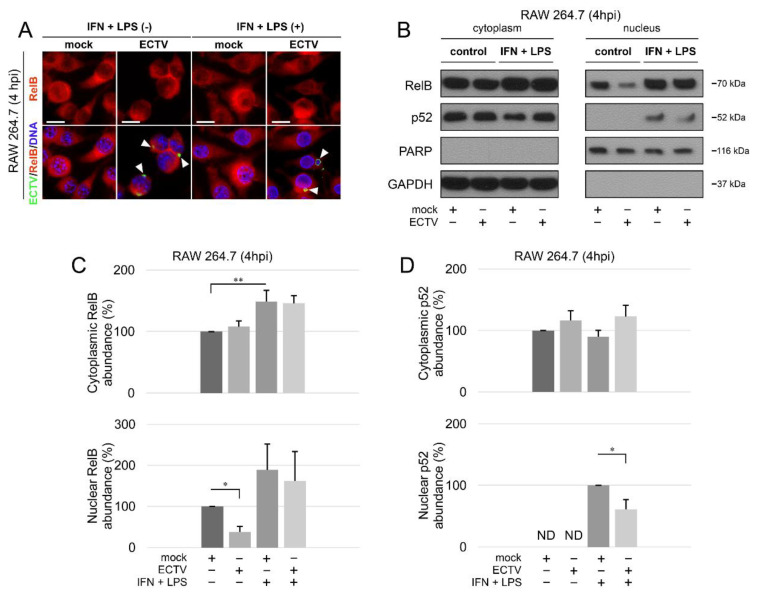
ECTV inhibits nuclear translocation of RelB and p52 in RAW 264.7 cells at 4 hpi. (**A**) Immunofluorescence microscopy images of cellular distribution of RelB in mock- and ECTV-infected RAW 264.7 cells. Cells were either unstimulated or stimulated with rmIFN-γ + *Escherichia coli* LPS O111:B4. RelB—red fluorescence; ECTV antigens—green fluorescence; DNA—blue fluorescence. White arrowheads indicate viral factories. Scale bar—20 μm. (**B**) Immunoblot analysis of RelB and p52 content in cytoplasmic and nuclear fractions of mock- and ECTV-infected RAW 264.7 cells. The cells were either unstimulated or were stimulated with rmIFN-γ + LPS O111:B4. GAPDH—cytoplasmic loading control, PARP—nuclear loading control. (**C**) Densitometric quantification of RelB abundance in cytoplasmic and nuclear fractions of mock- and ECTV-infected RAW 264.7 cells. (**D**) Densitometric quantification of p52 content in cytoplasmic and nuclear fractions of mock- and ECTV-infected RAW 264.7 cells. The data were obtained from three independent biological experiments. * *p* ≤ 0.05, ** *p* ≤ 0.01.

According to our results, ECTV diminished the nuclear content of RelB (Figure 2A–C). However, while the stimulation of cells with IFN-γ + LPS alone led to a significant increase in the content of cytoplasmic RelB only, the increase in the nuclear content of RelB was not statistically significant (Figure 2C). In addition, we detected nuclear p52 in cells treated with IFN-γ + LPS. Furthermore, in ECTV-infected cells treated with IFN-γ + LPS, nuclear p52 content was downregulated (Figure 2B,D). These data suggest that ECTV inhibits RelB/p52 activation at an early stage of the replication cycle, during which the viral factories start to appear.

### 3.2. ECTV Counteracts PMA/Io-, LPS-, and IFN-Γ + LPS-Induced RelB Nuclear Translocation in JAWS II and RAW 264.7 Cells at 12-24 hpi

According to the literature, RelB is activated after the treatment of cells with the inducers of the canonical NF-κB signaling pathway, such as LPS [36,37] and PMA + Io [38]. Therefore, we further examined the effect of ECTV on JAWS II and RAW 264.7 cells treated with LPS, PMA/Io, and IFN-γ + LPS to evaluate the nuclear translocation of RelB. However, CD40L, which stimulates the noncanonical NF-κB signaling in DCs and macrophages [34], was not used in our work due to the low expression of CD40 in both the tested cell lines [33,39]. 

**Figure 3:**
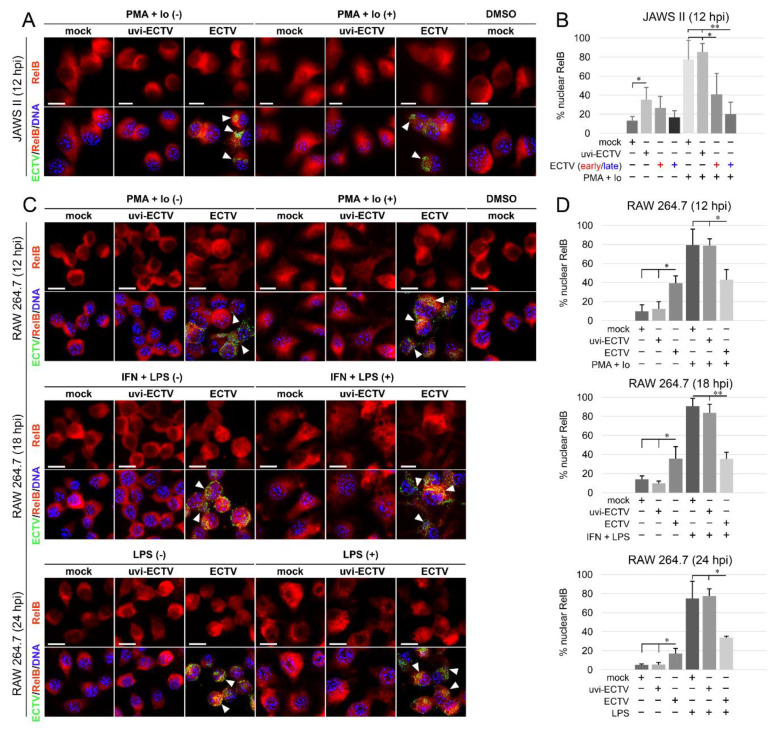
ECTV influences nuclear translocation of RelB in JAWS II and RAW 264.7 cells at 12–24 hpi. (**A**) Immunofluorescence microscopy images of subcellular localization of RelB in control and PMA + Io-treated mock-, uvi-ECTV, and ECTV-infected JAWS II cells at 12 hpi. RelB—red fluorescence, ECTV antigens—green fluorescence, DNA—blue fluorescence. DMSO—solvent control. White arrowheads indicate viral factories. Scale bar—20 μm. (**B**) Quantification of nuclear RelB-positive cells. Mock-, uvi-ECTV, and ECTV-infected JAWS II cells displaying round (early) and bloated (late) viral factories were analyzed. Graphs represent the percentage of cells displaying a greater intensity of fluorescence in the nucleus compared to control cells. Data were obtained from three independent experiments. A total of 50 cells/condition/experiment was counted (* *p* ≤ 0.05, ** *p* ≤ 0.01). (**C**) Immunofluorescence analysis of RelB subcellular localization in mock-, uvi-ECTV-, and ECTV-infected RAW 264.7 cells. Cells were either unstimulated or stimulated with PMA + Io, rmIFN-γ + *Escherichia coli* LPS O111:B4, or LPS O111:B4 and analyzed at 12, 18, and 24 hpi, respectively. (**D**) Quantification of nuclear translocation of RelB in mock-, uvi-ECTV, and ECTV-infected RAW 264.7 cells with bloated viral factories. Graphs represent the mean percentage of RelB-positive cells from three independent experiments. A total of 100 cells/condition/experiment was analyzed (* *p* ≤ 0.05, ** *p* ≤ 0.01).

Analysis of the subcellular localization of RelB in mock-, uvi-ECTV-, and ECTV-infected JAWS II cells revealed that compared to mock-infected cells, there was a significant increase in the percentage of RelB-positive cells after treatment of the cells with uvi-ECTV. It was also noticed that ECTV counteracted the RelB nuclear accumulation induced by PMA + Io. While around 80% of the cells were stimulated after PMA + Io treatment, around 40% were stimulated at early the viral replication stage, and 20% were stimulated at the late viral replication stage (Figure 3A,B). At 12, 18, and 24 h after ECTV infection in RAW 264.7 cells, a significant increase in the percentage of cells with nuclear accumulation of RelB was observed; however, RelB was activated in less than 40% of the infected cells. We also observed the domination of late viral factories starting from 12 hpi (Figure 3C,D). After treatment of the cells with PMA + Io, IFN-γ + LPS, or LPS alone, we observed, respectively, around 80%, 90%, and 75% of the cells displaying nuclear accumulation of RelB. Meanwhile, ECTV inhibited the effect of PMA + Io (around 45% of positive cells) and IFN-γ + LPS and LPS alone (around 35% of positive cells) (Figure 3D). These findings indicate that ECTV actively counteracts the nuclear translocation of RelB induced by both inducers of canonical and noncanonical NF-κB signaling.

### 3.3. ECTV Inhibits Phosphorylation of RelB and p100 and Affects p100/p52 Content in JAWS II and RAW 264.7 Cells

To further confirm the impact of ECTV on the activation of the noncanonical NF-κB signaling components, we determined the changes in the level of phosphorylation of RelB and p100. It has been shown that PMA + Io induces phosphorylation of RelB at Thr84 and Ser552 and its subsequent degradation in T cells [40]. Since we hypothesized that ECTV affects the level of phosphorylation of both RelB and p100, we assessed the level of phosphorylation of RelB at Ser552 and p100 phosphorylation at Ser872 upon ECTV infection of JAWS II DCs and RAW 264.7 macrophages.

**Figure 4:**
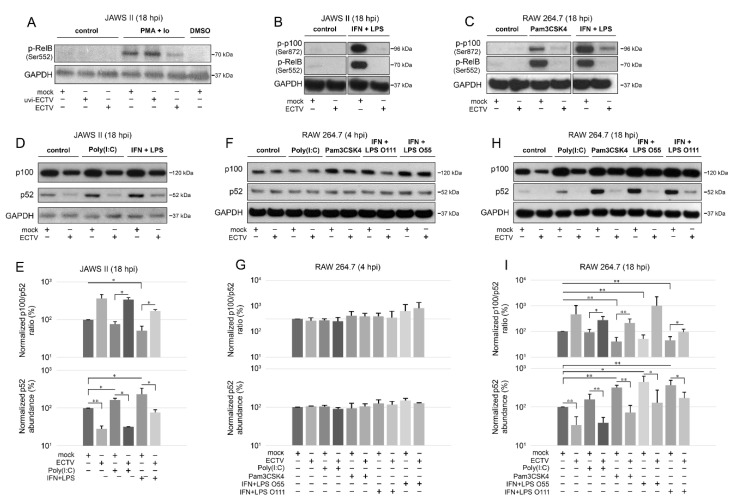
ECTV inhibits phosphorylation of p100 and RelB and affects p100 processing in JAWS II and RAW 264.7 cells. (**A**) Immunoblot analysis of phosphorylation of RelB in unstimulated or PMA + Io-stimulated mock-, uvi-ECTV-, and ECTV-infected JAWS II cells. (**B**) Immunoblot analysis of the content of p-RelB and p-p100 in unstimulated or rmIFN-γ *+ Escherichia coli* O111:B4 LPS-stimulated mock- and ECTV-infected JAWS II cells. (**C**) Immunoblot analysis of p-RelB and p-p100 in unstimulated and Pam3CSK4- or rmIFN-γ + LPS O111:B4-stimulated mock- and ECTV-infected RAW 264.7 cells. (**D**) Immunoblot analysis of p100/p52 content in mock- and ECTV-infected JAWS II cells treated with poly(I:C) or rmIFN-γ + LPS O111:B4. (**E**) Densitometric analysis of the p100/p52 ratio and p52 content in JAWS II cells. Data obtained from two independent experiments are shown on histograms with a logarithmic scale. * *p* ≤ 0.05, ** *p* ≤ 0.01. GAPDH—loading control. (**F**) Immunoblot analysis of p100/p52 proteins in mock- and ECTV-infected RAW 264.7 cells at 4 hpi. The cells were left untreated or treated with poly(I:C), Pam3CSK4, rmIFN-γ + *E. coli* LPS O55:B5, or rmIFN-γ + LPS O111:B4. (**G**) Densitometric analysis of the p100/p52 ratio and p52 content in RAW 264.7 cells based on the data obtained from two independent experiments. Data are presented on histograms with a logarithmic scale. GAPDH—loading control. (**H**) Immunoblot analysis of p100/p52 proteins in mock- and ECTV-infected RAW 264.7 cells at 18 hpi. The cells were left untreated or treated with poly(I:C), Pam3CSK4, rmIFN-γ + E. coli LPS O55:B5, or rmIFN-γ + LPS O111:B4. (**I**) Densitometric analysis of the p100/p52 ratio and p52 content in RAW 264.7 cells based on the data obtained from two independent experiments. Data are presented on histograms with a logarithmic scale. * *p* ≤ 0.05, ** *p* ≤ 0.01. GAPDH—loading control.

Our results showed that ECTV blocked the phosphorylation of both RelB (Ser552) and p100 (Ser872) that was induced by Pam3CSK4, PMA + Io, or IFN-γ + LPS (Figure 4A–C). Since p100 Ser872 phosphorylation by IKKα serves its ubiquitination [41], we hypothesized that ECTV also prevents the activity of p52.

Next, we evaluated the ratio of p100/p52 after ECTV infection. LPS derived from different bacterial strains differently induces the innate immune response in macrophages; therefore, for macrophage treatment, we used LPS derived from *E. coli* O55:B5 and O111:B4 strains [42]. Both cell lines were induced with poly(I:C) that acts via TLR3 to activate NF-κB2 [43]. The cells were also treated with Pam3CSK4 to activate noncanonical NF-κB signaling [7]. 

After 18 h of stimulation, there was a significant decrease in the ratio of p100/p52 in JAWS II cells induced by IFN-γ + LPS. ECTV infection, in turn, significantly increased the ratio of p100/p52 in poly(I:C)- or IFN-γ + LPS-treated cells (Figure 4D,E). Importantly, both ECTV alone and combined with all the ligands used for the stimulation significantly decreased the p52 level in JAWS II cells (Figure 4E). In RAW 264.7 macrophages, we did not observe any significant difference in the ratio of p100/p52 at 4 hpi (Figure 4F,G). At 18 hpi, a significant decrease in the ratio of p100/p52 was observed after treating the cells with Pam3CSK4 or IFN-γ + LPS. In addition, a significant increase in the ratio of p100/p52 was observed in ECTV-infected cells that were treated with poly(I:C), Pam3CSK4, or IFN-γ + LPS O111:B4 but not with IFN-γ + LPS O55:B5. However, ECTV decreased p52 levels in both unstimulated and stimulated cells (Figure 4H,I). 

These results show that ECTV influences NF-κB2 in JAWS II DCs and RAW 264.7 macrophages, thereby affecting noncanonical NF-κB signaling components at later stages of viral replication.

### 3.4. ECTV Influences the Cellular Level of Components of the Noncanonical NF-κB Signaling Pathway at Late Stages of Viral Lifecycle

**Figure 5:**
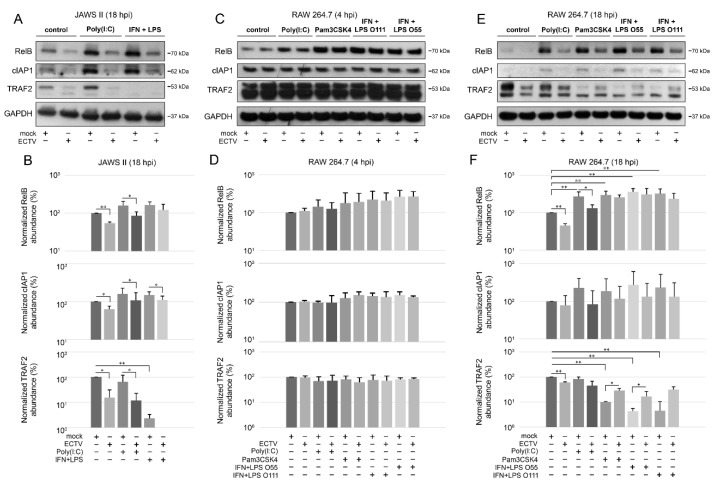
ECTV influences the expression of components of the noncanonical NF-κB signaling pathway in JAWS II and RAW 264.7 cells. (**A**) Immunoblots of mock- and ECTV-infected JAWS II cells untreated or treated with poly(I:C) or rmIFN-γ *+ Escherichia coli* LPS O111:B4. (**B**) Densitometric evaluation of RelB, cIAP1, and TRAF2 expression in JAWS II cells. The analysis was based on the data of two independent biological experiments. The data are shown on histograms with a logarithmic scale. * *p* ≤ 0.05, ** *p* ≤ 0.01. GAPDH—loading control. (**C**) Immunoblots of mock- and ECTV-infected RAW 264.7 cells at 4 hpi. Cells were left untreated or were treated with poly(I:C), Pam3CSK4, rmIFN-γ + *E. coli* LPS O55:B5, or rmIFN-γ + LPS O111:B4. (**D**) Densitometric evaluation of RelB, cIAP1, and TRAF2 expression in RAW 264.7 cells. The analysis was based on the data of two independent biological experiments and is plotted on histograms with a logarithmic scale. GAPDH—loading control. (**E**) Immunoblots of mock- and ECTV-infected RAW 264.7 cells at 18 hpi. Cells were left untreated or were treated with poly(I:C), Pam3CSK4, rmIFN-γ + *E. coli* LPS O55:B5, or rmIFN-γ + LPS O111:B4. (**F**) Densitometric evaluation of RelB, cIAP1, and TRAF2 expression in RAW 264.7 cells. The analysis was based on the data of two independent biological experiments and is plotted on histograms with a logarithmic scale. * *p* ≤ 0.05, ** *p* ≤ 0.01. GAPDH—loading control.

To further investigate the effect of ECTV on the noncanonical NF-κB signaling components, we evaluated their cellular level during ECTV infection using Western blot analysis. We initially examined the expression of NIK kinase in RAW 264.7 cells at 4 and 18 hpi and found that there were no significant changes in the ECTV-infected cells. However, our preliminary data showed that ECTV inhibits the expression of TRAF3, a negative regulator of noncanonical NF-κB signaling, at 4 and 18 hpi (Figure S1). Therefore, we assumed that ECTV may alter the expression of the other components involved in the noncanonical NF-κB activation pathway. We observed that in JAWS II cells, ECTV significantly decreased the content of RelB, cIAP1, and TRAF2 at 18 hpi. Following stimulation of JAWS II cells with IFN-γ + LPS O111:B4, a significant decrease in TRAF2 content was detected. In addition, ECTV infection resulted in a significant decrease in the level of TRAF2, RelB, and cIAP1 in cells treated with poly(I:C). Upon IFN-γ + LPS O111:B4 treatment, only cIAP1 level was significantly lower in ECTV-infected cells (Figure 5A,B). In ECTV-infected RAW 264.7 cells, no significant changes in the analyzed protein content were observed at 4 hpi (Figure 5C,D). At 18 hpi, a significant decrease in the protein level was observed for RelB and TRAF2, but not for cIAP1. In turn, RelB level increased upon cell treatment with poly(I:C), Pam3CSK4, IFN-γ + LPS O55:B5, and IFN-γ + LPS O111:B4. However, ECTV actively inhibited the expression of RelB after stimulation with poly(I:C). The level of TRAF2, in turn, was significantly reduced in the cells treated with Pam3CSK4, IFN-γ + LPS O55:B5, and IFN-γ + LPS O111:B4. In addition, ECTV significantly increased TRAF2 levels in cells stimulated with Pam3CSK4 and IFN-γ + LPS O55:B5 (Figure 5E,F). These results show that the inhibition of antiapoptotic cIAP1, RelB, and TRAF2 in both types of cells occurs at the later stages of infection (18 hpi), when apoptosis and release of progeny virions complete the virus replication cycle [31,44]. More importantly, protein expression of TRAF2 was significantly low in both cell lines after stimulation with IFN-γ + LPS. The effect of ECTV on the expression of TRAF2 was significant after stimulation with poly(I:C) in JAWS II cells but not in RAW 264.7 macrophages. While in JAWS II cells the inhibitory effects of ECTV on lowered TRAF2 level in IFN-γ + LPS-treated cells were not statistically significant (Figure 5B), in RAW 264.7 cells, the tendency was the opposite but was statistically significant for LPS O55:B5 (Figure 5F). Taken together, ECTV exhibits different modes of action on infected JAWS II and RAW 264.7 cells after NF-κB stimulation.

### 3.5. ECTV Inhibits RelB and p52 DNA-Binding Activity

**Figure 6:**
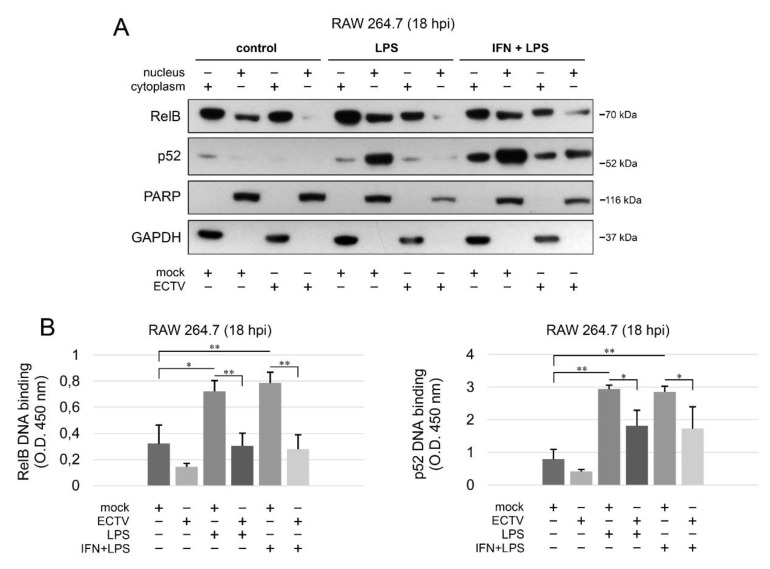
ECTV counteracts nuclear translocation and DNA binding by RelB and p52. (**A**) Immunoblot analysis of the content of RelB and p52 in cytoplasmic and nuclear fractions of mock- and ECTV-infected RAW 264.7 cells unstimulated or stimulated with *Escherichia coli* LPS O111:B4 alone or rmIFN-γ + LPS O111:B4 for 18 h. GAPDH—cytoplasmic loading control, PARP—nuclear loading control. (**B**) DNA-binding ELISA of RelB and p52. The 5′-GGGACTTTCC-3′ oligonucleotide binding by RelB and p52 was evaluated in nuclear extracts of mock- and ECTV-infected RAW 264.7 cells. The results of the analysis of DNA binding displayed as OD 450 nm values of the analyzed extracts were based on three independent experiments (* *p* ≤ 0.05, ** *p* ≤ 0.01).

Since we observed the inhibition of RelB nuclear translocation and diminished p52 content in ECTV-infected cells after stimulation with various ligands at 18 hpi, we further investigated the effect of ECTV on the transcriptional activity of RelB and p52. First, we analyzed the level of RelB and p52 protein in cytoplasmic and nuclear fractions of RAW 264.7 cells by Western blot analysis. While LPS alone and IFN-γ + LPS increased the level of RelB and p52 in the nucleus of mock-infected RAW 264.7 cells, ECTV counteracted this effect (Figure 6A). To measure the DNA-binding activity by RelB and p52, we conducted DNA-binding ELISA using the nuclear extracts obtained from RAW 264.7 macrophages. We observed that LPS and IFN-γ + LPS significantly increased the binding of 5′-GGGACTTTCC-3′ oligonucleotide by RelB and p52. ECTV, in turn, did not significantly affect the DNA binding compared to the mock-infected cells but actively inhibited the binding of the oligonucleotide by RelB and p52 in the cells stimulated with LPS or IFN-γ + LPS (Figure 6B). Thus, we confirmed that ECTV represses the transcriptional activity of RelB and p52, triggered by canonical and noncanonical NF-κB inducers.

### 3.6. ECTV Inhibits the Expression of Genes Related to Noncanonical NF-κB Signaling

**Figure 7:**
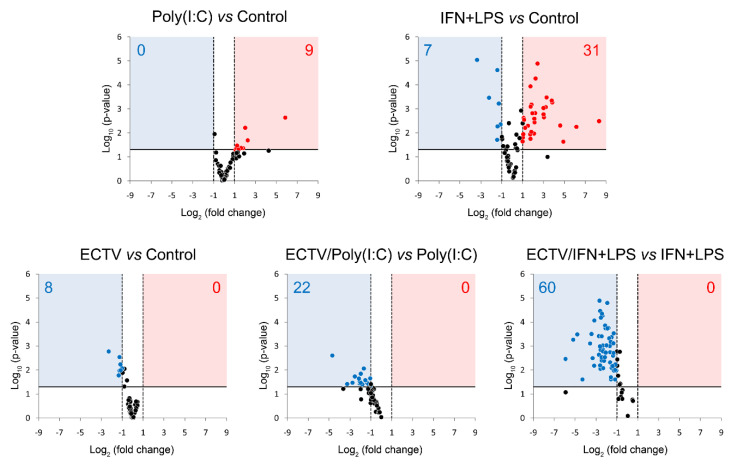
ECTV downregulates the expression of genes involved in NF-κB signaling. The expression of the analyzed genes was evaluated using RT-qPCR. Volcano plot graphs show the differences in the activation of NF-κB signaling at transcriptional level in mock- and ECTV-infected RAW 264.7 macrophages, untreated or treated with poly(I:C) or rmIFN-γ + *Escherichia coli* LPS O111:B4 for 18 h. The relative expression of the analyzed transcripts is represented by the *X*-axis. Changes in gene expression are shown in different colors (red–upregulation, blue—downregulation, black—less than two or nonsignificant change). Fold change cutoff is shown by vertical dotted lines, whereas *p*-value cutoff is represented by a horizontal solid line. Data were obtained from three independent experiments (*p* ≤ 0.05).

Our last question concerned the effect of ECTV on the expression of genes encoding the components of the noncanonical NF-κB signaling pathway. Due to the existing cross-talk between the two NF-κB signaling pathways, we investigated the effect of their inducers on the expression of the genes encoding the noncanonical NF-κB components and other genes related to noncanonical NF-κB signalling (Figure 7, Tables S1–S5). 

After 18 h of poly(I:C) treatment, we observed the upregulation of 9 genes related to the noncanonical NF-κB signaling (Figure 7, Table S1). However, after 18 h of IFN-γ + LPS treatment, there was an upregulation and downregulation of the expression of 31 and 7 genes, respectively, that were related to the noncanonical NF-κB signaling pathway (Figure 7, Table S2). More importantly, while ECTV alone downregulated 8 out of the 86 analyzed genes (Figure 7, Table S3), it actively inhibited the expression of 22 and 60 genes in the cells stimulated with poly(I:C) and IFN-γ + LPS, respectively (Figure 7, Tables S4 and S5). 

**Figure 8:**
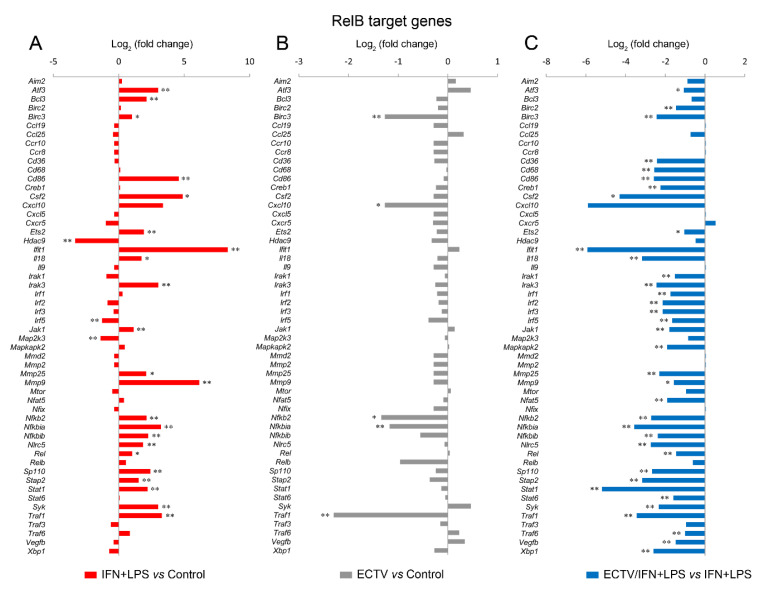
ECTV inhibits the expression of RelB target genes in RAW 264.7 macrophages upon the induction of RelB nuclear translocation. The expression of the analyzed genes was evaluated using RT-qPCR. (**A**) The effect of the stimulation of RAW 264.7 cells with rmIFN-γ + *Escherichia coli* LPS O111:B4 for 18 h on RelB-controlled genes. (**B**) The influence of ECTV (18 hpi) on RelB target genes. (**C**) The impact of ECTV on the expression of RelB-dependent genes following RAW 264.7 stimulation with rmIFN-γ + LPS O111:B4 for 18 h. Data represent three independent experiments (* *p* ≤ 0.05, ** *p* ≤ 0.01).

**Figure 9:**
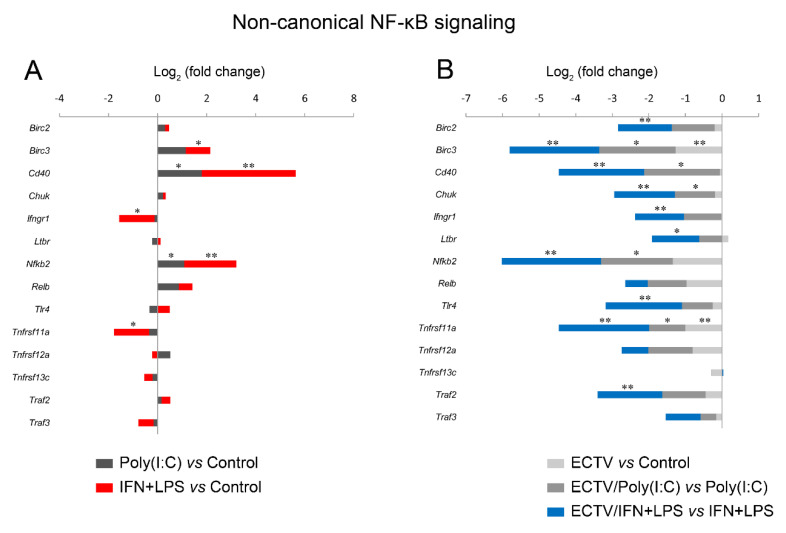
ECTV downregulates the genes of the crucial components of the noncanonical NF-κB signaling pathway. The expression of the analyzed genes was evaluated using RT-qPCR. (**A**) The influence of poly(I:C) and rmIFN-γ + *Escherichia coli* LPS O111:B4 on the expression of the genes encoding noncanonical NF-κB signaling components. (**B**) The changes in the expression of the genes related to the noncanonical NF-κB signaling pathway in unstimulated and stimulated RAW 264.7 cells upon ECTV infection. Data were derived from three independent experiments (* *p* ≤ 0.05, ** *p* ≤ 0.01).

Since IFN-γ + LPS induced the nuclear translocation of RelB in RAW 264.7 cells, we expected that RelB target genes will be upregulated upon the stimulation of cells with these activators. To select the target genes of RelB, we used the TRANSFAC database of eukaryotic transcription factors [45]. After stimulation of RAW 264.7 cells with IFN-γ + LPS, we observed a significant upregulation of the following RelB target genes—*Atf3*, *Bcl3*, *Birc3*, *Cd86*, *Csf2*, *Ets2*, *Ifit1*, *Il18, Irak3*, *Jak1*, *Mmp9*, *Mmp25*, *Nfkb2*, *Nfkbia*, *Nfkbib*, *Nlrc5*, *Rel*, *Sp110*, *Stap2*, *Stat1*, *Syk*, and *Traf1* (Figure 8A). In addition, IFN-γ + LPS treatment resulted in the downregulation of *Hdac9*, *Irf5*, and *Map2k3* (Figure 8A). In ECTV-infected cells, in turn, downregulation of *Birc3*, *Cxcl10*, *Nfkb2*, *Nfkbia*, and *Traf1* was observed (Figure 8B). However, ECTV downregulated *Atf3*, *Birc2*, *Birc3*, *Cd36*, *Cd68*, *Cd86*, *Creb1*, *Csf2*, *Ets2*, *Ifit1*, *Il18*, *Irak1*, *Irak3*, *Irf1*, *Irf2*, *Irf3*, *Irf5*, *Jak1*, *Mapkapk2*, *Mmp9*, *Mmp29*, *Nfat5*, *Nfkb2*, *Nfkbia*, *Nfkbib*, *Nlrc5*, *Rel*, *Sp110*, *Stap2*, *Stat1*, *Stat6*, *Syk*, *Traf1*, *Traf6*, *Vegfb*, and *Xbp1* genes in IFN-γ + LPS-treated cells (Figure 8C). Next, we selected the genes encoding the major components of the noncanonical NF-κB signaling pathway and compared their mRNA expression in unstimulated versus poly(I:C)-treated cells and IFN-γ + LPS versus control cells. The role of poly(I:C) in the regulation of the noncanonical NF-κB signaling is, as previously mentioned, based on NF-κB2 activation [35]. However, our previous study has shown that the long-term stimulation of RAW 264.7 cells with poly(I:C) results in the nuclear translocation of RelA [24]. We observed that poly(I:C) upregulated the expression of *Cd40* and *Nfkb2*, which are target genes of RelA [46], whereas IFN-γ + LPS upregulated the expression of *Birc3*, *Cd40*, and *Nfkb*2 and downregulated the expression of *Ifngr1* and *Tnfrsf11a* (Figure 9A). ECTV, in turn, downregulated the expression of *Birc3* and *Tnfrsf12a*. In poly(I:C)-treated cells, ECTV infection resulted in the inhibition of *Birc3*, *Cd40*, *Chuk*, *Nfkb2*, and *Tnfrsf11a*. In IFN-γ + LPS-treated cells, in turn, ECTV inhibited the expression of *Birc2*, *Birc3*, *Cd40*, *Chuk*, *Ifngr1*, *Ltbr*, *Nfkb2*, *Tlr4*, *Tnfrsf11a*, and *Traf2* (Figure 9B). These results suggest that the function of the components of the noncanonical NF-κB signaling pathway is disrupted by ECTV.

**Figure 10:**
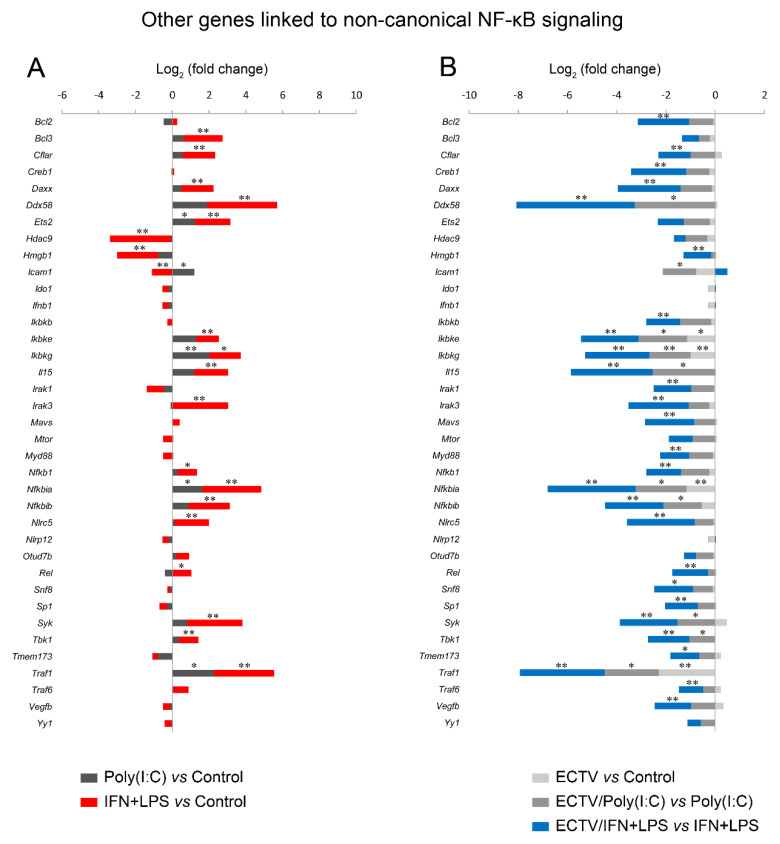
ECTV inhibits the expression of the genes associated with the noncanonical NF-κB signaling. The expression of the analyzed genes was evaluated using RT-qPCR. (**A**) Influence of poly(I:C) and rmIFN-γ + *Escherichia coli* LPS O111:B4 on genes linked to the noncanonical NF-κB activation pathway in RAW 264.7 macrophages following 18 h of cell stimulation. (**B**) Comparison of mock- and ECTV-infected RAW 264.7 cells after stimulation with poly(I:C) or rmIFN-γ + LPS O111:B4 at 18 hpi. Data were acquired from three independent experiments (* *p* ≤ 0.05, ** *p* ≤ 0.01).

Next, we analyzed the influence of ECTV on genes that are associated with the noncanonical NF-κB signaling. We observed that poly(I:C) treatment resulted in the upregulation of *Ets2*, *Icam1*, *Ikbkg*, *Nfkbia*, and *Traf1*, which are regulated by RelA [46]. ECTV alone inhibited the expression of *Ikbke*, *Ikbkg*, and *Nfkbia* genes. However, in the cells treated with poly(I:C), ECTV actively downregulated the expression of *Ddx58*, *Icam1*, *Ikbke*, *Ikbkg*, *Il15*, *Nfkbia*, *Nfkbib*, *Syk*, *Tbk1*, and *Traf1*. We also observed that the expression of numerous genes encoding proteins that may affect the noncanonical NF-κB signaling was modulated by IFN + LPS and actively downregulated by ECTV (Figure 10).

These results confirm that in macrophages, IFN-γ + LPS potently influences RelB target genes and other genes linked to NF-κB, whereas ECTV actively inhibits the expression of numerous genes after stimulation of RAW 264.7 macrophages with poly(I:C) or IFN + LPS.

## 4. Discussion

In recent years, there has been growing evidence that noncanonical NF-κB signaling regulates the innate immune response and controls the activation of the canonical NF-κB signaling pathway. In particular, NIK, the crucial component of the noncanonical NF-κB signaling pathway, negatively regulates virus- and TLR-driven IFN-I induction, thereby regulating antiviral innate immunity [11]. 

Since ECTV productively infects both RAW 264.7 murine macrophages and JAWS II immature DCs [28,47], we used these two cell lines as in vitro models for studying the noncanonical NF-κB signaling components in APCs, which link innate and adaptive immunity and initiate the antiviral immune response. The phenotype of JAWS II cell line derived from p53^−/−^ C57BL/6 mice is characterized as more homogeneous compared to bone marrow-derived DCs (BMDCs). Therefore, JAWS II cells can be more useful for studying immunogenicity and proinflammatory responses [39,48]. However, JAWS II cells lack p53; therefore, they are more protected from apoptosis in cell culture. In turn, p53, which displays both pro- and antiapoptotic functions depending on the cellular context, may either cooperate with NF-κB or compete for cofactor proteins [49,50].

Interactions between *Poxviridae* family members and canonical NF-κB signaling are well described [51]. Our previous work showed the influence of ECTV on canonical NF-κB signaling and inflammatory response in RAW 264.7 cells [29]. We have also previously demonstrated that in GM-CSF-derived bone marrow (GM-BM) cells, ECTV inhibits nuclear translocation of RelA, downregulates the expression of genes involved in cell signaling including *Relb*, and impedes cytokine and chemokine responses [28,52]. Because both canonical and noncanonical NF-κB signaling pathways cooperate, we hypothesized that ECTV affects the noncanonical NF-κB signaling in a similar fashion. To further delineate the influence of ECTV on NF-κB in APCs, we focused on the components of the noncanonical NF-κB signaling pathway. Certain data indicate the influence of poxviruses on the components of the noncanonical NF-κB activation pathway. The member of genus *Parapoxvirus*, ORFV encodes ORFV073 protein that inhibits a regulatory kinase IKKγ and may affect TRAF2 [53], which is necessary for IFN-induced noncanonical NF-κB signaling [54,55]. Another study has shown that TRAF2 boosts the replication of VACV [56]. More importantly, TRAF2 may be reduced in proteasomes upon TLR stimulation; therefore, it is likely that ECTV, which encodes proteasome inhibitors [23,24,25,26], may influence TRAF2 processing. It has been shown that in bone marrow-derived macrophages (BMDMs) treated with Pam3CSK4, both cIAP1 and TRAF2 undergo proteasomal reduction [57]. The inhibitory effect of ECTV on cIAP1 and TRAF2 in JAWS II cells suggests that inhibition of cIAP1 and TRAF2 serves apoptosis execution in p53^−/−^ BMDCs by ECTV. In RAW 264.7 cells, in turn, the downregulation of cIAP1 depends on nitric oxide (NO) and correlates with apoptosis induced by IFN-γ + LPS [58]. In addition, cIAP1 is involved in the degradation of TRAF2 after the differentiation of monocytes into macrophages and maintains a low level of TRAF2 in differentiated macrophages [59]. We hypothesize that the inhibition of expression of *Birc3* and cIAP1 by ECTV at 18 hpi, which was observed after cell stimulation with poly(I:C) or IFN-γ + LPS, may induce apoptosis at later stages of infection. Our data also suggest a different mode of regulation for TRAF2 in macrophages and p53-deficient JAWS II cells. TRAF2 is considered as a negative regulator of the noncanonical NF-κB signaling, and TRAF2-deficient macrophages are characterized by NIK accumulation and p100 processing. Moreover, TRAF2 has an anti-inflammatory function, which is independent of NIK [60]. Therefore, we hypothesize that ECTV prevents the loss of TRAF2 to maintain the anti-inflammatory state of the cells. It is also worth noticing that although the expression of *Traf2* gene was inhibited by ECTV in the cells treated with LPS+IFN, the level of TRAF2 protein was higher in the cells treated with ECTV upon stimulation with LPS+IFN, whereas this tendency was not observed in the cells treated with poly(I:C). Since poly(I:C) induces canonical NF-κB signaling through TLR3, while IFN+LPS triggers the noncanonical NF-κB pathway, ECTV may affect these two cellular pathways differentially. It has been shown that in RAW 264.7 macrophages, IFN enhances the response of macrophages to LPS and NF-κB activation as well as hyperstimulates cytokine response [61]. We assume that ECTV may induce the accumulation of TRAF2 to counteract the enhanced induction of inflammatory response. Interestingly, studies on VACV postulate that certain cellular mRNAs prevent mRNA depletion and translational repression during infection, in order to maintain cellular integrity, which is required for viral replication. This phenomenon was observed in the case of proteins involved in oxidative phosphorylation in VACV-infected cells when there was a need for the production of cellular energy. Although the mRNA levels of the proteins involved in oxidative phosphorylation were downregulated, the protein levels were upregulated upon VACV infection. It was assumed that short 5′ untranslated regions (5′UTRs) and coding DNA sequences (CDSs) that resemble VACV transcripts may be involved in translation during the host shutoff [62]. It cannot be excluded that similar mechanisms may exist in ECTV-infected cells.

Since we also focused on the evaluation of activation of RelB and p100, we used PMA + Io to induce the phosphorylation of RelB and p100. PMA + Io phosphorylates RelB at Ser552 and Thr84 and triggers degradation of RelB in Jurkat cells and human peripheral blood T cells [63], which is regulated by glycogen synthase kinase (GSK)-3β [64]. We hypothesize that the active inhibition of PMA + Io-induced phosphorylation events by ECTV interferes with GSK-3β activity.

In this study, we also focused on the activation of RelB by canonical and noncanonical NF-κB stimuli. Our results agree with those of other studies, which have also demonstrated that in RAW 264.7 macrophages and BMDMs, RelB is activated by LPS. RelA, rapidly stimulated by LPS and TNF, activates the transcription of *Relb* gene. Delayed nuclear translocation of RelB, in turn, correlates with increased transcription of *Relb* gene. More importantly, both RelA and RelB regulate the transcription of the RelB-encoding gene [36,65]. 

In our experiment, RAW 264.7 cells showed nuclear accumulation of p52 after 4 h of incubation of cells with IFN-γ + LPS. It has been demonstrated that in RAW 264.7 cells treated with IFN-γ + LPS, p52 binds to inducible NO synthase (iNOS) and cytochrome c oxidase (COX) promoters after 8 h of cell stimulation [35]. Furthermore, IFN-γ + LPS triggers apoptosis in RAW 264.7 macrophages by the activation of iNOS and nitrite accumulation [66]. While ECTV inhibited the IFN-γ + LPS-induced nuclear accumulation of p52 at 4 hpi, as well as expression of *Nfkb2* gene and activation of both RelB and p52 at 18 hpi, we cannot exclude that the changes in the levels of cIAP1 and TRAF2 proteins induced by ECTV infection upon IFN-γ + LPS stimulation may facilitate apoptosis in ECTV-infected RAW 264.7 macrophages at later stages of infection. 

The noncanonical NF-κB signaling regulates antiviral innate immunity via the inhibition of IFN-I expression by RelB and p52 [11]. In this study, we demonstrated that IFN signaling can be disrupted by ECTV, which inhibited the expression of *Ifngr1* receptor, *Irf5* transcription factor, and *Ifit1* belonging to IFN-stimulated genes (ISGs). More importantly, the latter two are controlled by RelB. It is noteworthy that IRF5 regulates macrophage polarization and proinflammatory activation of macrophages [67,68], whereas in myeloid DCs, IRF5 controls the response of IFN-I and activation of ISGs [69]. When stimulated by IFN-I, ISGs may act as activators of macrophages, which, in turn, activate B and T cells [70,71]. Since macrophages are involved in the activation of adaptive immunity, ISG inhibition by ECTV may disrupt the induction of both innate and adaptive immune responses. In addition, ECTV and other poxviruses, including VACV, encode cap-specific mRNA (nucleoside-2′-O-)-methyltransferase. It is believed that Ifit1, a protein expressed by the host cell, may prevent the translation of viral mRNA by binding to 2′-O-methyl-deficient capped RNAs, whereas 2′-O-methylation of mRNA serves viral evasion [72,73]. Since Ifit1 inhibits the synthesis of viral proteins, we assume that the inhibition of expression of *Ifit1* gene by ECTV counteracts antiviral response.

When studying the modulatory mechanisms of ECTV, we compared the effect of the virus on different TLR agonists activating the components of the NF-κB noncanonical signaling pathway. It has been shown that the stimulation of BMDCs with Pam3CSK4 induces the noncanonical NF-κB signaling and DNA–RelB binding [7]. Poly(I:C) triggers the expression of NF-κB2 p100/p52, p52-dependent transactivation and activates RelA [43], and stimulates nuclear translocation of RelB in murine BMDMs [74]. We found that ECTV diminished RelB expression induced by poly(I:C) but not by Pam3CSK4 or IFN-γ + LPS. These results demonstrate the different effects of orthopoxviruses on the expression and activation of the NF-κB family of transcription factors after treatment with various ligands.

## 5. Conclusions

In summary, our results clearly demonstrate that ECTV interferes with the activation of components of noncanonical NF-κB signaling after cell stimulation with various stimuli, including TLR ligands. This suggests that ECTV affects the noncanonical NF-κB signaling pathway components via inhibition of inflammatory signaling pathways linked to the canonical NF-κB activation. We assume that similar mechanisms may be exploited by orthopoxviruses in innate and adaptive immune cells, such as DCs and macrophages. Since the effect of poxviruses on the canonical NF-κB signaling is well known, further studies on viral manipulation of noncanonical NF-κB signaling, which is linked to the canonical NF-κB signaling pathway, will enable the identification of the potential targets of viral manipulation. 

## Supplementary Materials

The following are available online at https://www.mdpi.com/2076-0817/9/10/814/s1, Figure S1: analysis of the content of NIK and TRAF3 in the mock- and ECTV-infected RAW 264.7 macrophages; Table S1: expression of genes involved in NF-κB signaling pathways in RAW 264.7 macrophages stimulated with poly(I:C) for 18 h; Table S2: expression of genes involved in NF-κB signaling pathways in RAW 264.7 macrophages stimulated with IFN+LPS for 18 h; Table S3: expression of genes involved in NF-κB signaling pathways in RAW 264.7 macrophages infected with ECTV (18 hpi); Table S4: expression of genes involved in NF-κB signaling pathways in RAW 264.7 macrophages infected with ECTV and stimulated with poly(I:C) (18 hpi); Table S5: expression of genes involved in NF-κB signaling pathways in RAW 264.7 macrophages infected with ECTV and stimulated with IFN+LPS (18 hpi).
