# Supplementary material for: First Insight into the Modulation of Noncanonical NF-κB Signaling Components by Poxviruses in Established Immune-Derived Cell Lines: An In Vitro Model of Ectromelia Virus Infection
Source: Pathogens. 2020 Oct 4;9(10):814. doi: 10.3390/pathogens9100814 (PMC7599462; doi:10.3390/pathogens9100814)
Supplement: Supplementary file 1 [file pathogens-09-00814-s001.pdf]

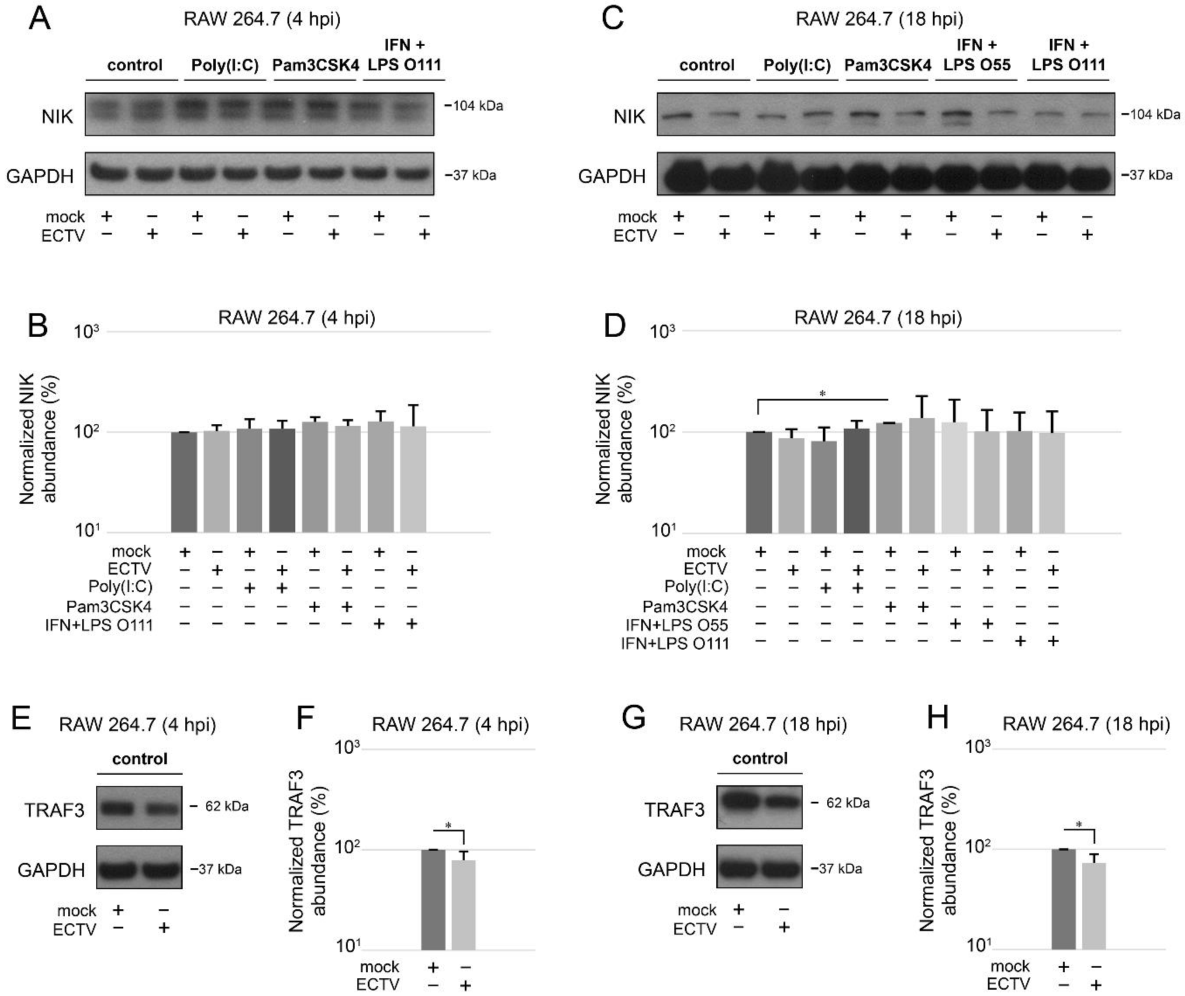

Supplementary Figure 1. Analysis of the content of NIK and TRAF3 in the mock- and ECTV-infected RAW 264.7 macrophages. (**A**) Immunoblot analysis of NIK content in mock- and ECTV-infected RAW 264.7 cells that were untreated or treated with poly(I:C), Pam3CSK4, or rmIFN-γ + *Escherichia coli* LPS O111:B4 at 4 hpi. (**B**) Densitometric evaluation of NIK expression in ECTV-infected RAW 264.7 cells (4 hpi). The analysis was based on the results of three independent biological experiments. The data are presented on a histogram with a logarithmic scale. GAPDH—loading control. (**C**) Immunoblot analysis of NIK content in the mock- and ECTV-infected RAW 264.7 cells that were untreated or treated with poly(I:C), Pam3CSK4, or rmIFN-γ + *E. coli* LPS O55:B5 or O111:B4 at 18 hpi. (**D**) Densitometric evaluation of NIK expression in ECTV-infected RAW 264.7 cells (18 hpi). The analysis was based on the results of two independent biological experiments. The data are presented on a histogram with a logarithmic scale. GAPDH—loading control (*$p \leq 0.05$). (**E**) Immunoblot analysis of TRAF3 content in the mock- and ECTV-infected RAW 264.7 cells at 4 hpi. (**F**) Densitometric evaluation of TRAF3 expression in ECTV-infected RAW 264.7 cells (4 hpi). The analysis was based on the results of two independent biological experiments. The data are presented on a histogram with a logarithmic scale. GAPDH—loading control (*$p \leq 0.05$). (**G**) Immunoblot analysis of TRAF3 content in the mock- and ECTV-infected RAW 264.7 cells at 18 hpi. (**H**) Densitometric evaluation of TRAF3 expression in ECTV-infected RAW 264.7 cells (18 hpi). The analysis was based on the results of two independent biological experiments. The data are presented on a histogram with a logarithmic scale. GAPDH—loading control (*$p \leq 0.05$).

Supplementary Table 1. Expression of genes involved in NF-κB signaling pathways in RAW 264.7 macrophages stimulated with poly(I:C) for 18 h.

	Poly(I:C) vs control		
**Gene**	**Fold change**	***p* value**	**Log_2 fold change**
Atf3	0,9727	0,837472	-0,039933177
Bcl2	0,7186	0,448858	-0,47673916
Bcl3	1,5227	0,170099	0,606631732
Birc2	1,2397	0,39966	0,309991039
Birc3	2,2191	0,073059	1,149974682
Ccl19	0,8685	0,521313	-0,203402246
Ccl25	0,8766	0,963013	-0,190009416
Ccr10	0,8685	0,521313	-0,203402246
Ccr8	0,8685	0,521313	-0,203402246
Cd36	0,5261	0,011111	-0,926591045
Cd40	3,4903	0,042535	1,803351045
Cd68	1,0619	0,737596	0,086647913
Cd86	0,8685	0,521313	-0,203402246
Cflar	1,4845	0,282632	0,569977093
Chuk	1,162	0,499438	0,216610069
Creb1	0,9659	0,952453	-0,050054261
Csf2	0,8685	0,521313	-0,203402246
Cxcl10	2,2346	0,112797	1,160016608
Cxcl5	0,8685	0,521313	-0,203402246
Cxcr5	1,0425	0,690566	0,060047384
Daxx	1,4077	0,255517	0,493339909
Ddx58	3,7668	0,070815	1,913339435
Ets2	2,3511	0,033747	1,233335903
Hdac9	0,9772	0,980521	-0,033274231
Hmgb1	0,5864	0,065331	-0,770042991
Icam1	2,2763	0,043682	1,186690707
Ido1	0,8685	0,521313	-0,203402246
Ifit1	19,2484	0,055576	4,266666623
Ifnb1	0,8685	0,521313	-0,203402246
Ifngr1	0,9223	0,872043	-0,116691997
Ikbkb	0,9885	0,928764	-0,016687129
Ikbke	2,4453	0,065553	1,290011472
Ikbkg	4,0278	0,006102	2,009992048
Il15	2,2868	0,068131	1,193330195
Il18	1,0718	0,700665	0,100035721
Il9	0,8685	0,521313	-0,203402246
Irak1	0,727	0,368887	-0,459972731
Irak3	0,9374	0,967064	-0,0932633
Irf1	0,9223	0,964208	-0,116691997
Irf2	0,9075	0,777673	-0,140030452
Irf3	0,9309	0,913952	-0,103301897
Irf5	0,984	0,911822	-0,023269779
Irf8	0,6507	0,190601	-0,619935541
Jak1	1,162	0,591372	0,216610069
Ltbr	0,8586	0,674539	-0,219941922
Map2k3	0,8351	0,423571	-0,25997913
Mapkapk2	1,2864	0,324814	0,363339312
Mavs	1,021	0,89367	0,029982866
Mmd2	0,8685	0,521313	-0,203402246
Mmp2	0,8685	0,521313	-0,203402246

Poly(I:C) vs control			
**Gene**	**Fold change**	***p* value**	**Log_2 fold change**
Mmp25	0,8685	0,521313	-0,203402246
Mmp9	57,4141	0,002279	5,843333179
Mtor	1,0234	0,880486	0,033370138
Myd88	1,0281	0,828426	0,039980598
Nfat5	0,9482	0,923404	-0,076736702
Nfix	0,8685	0,521313	-0,203402246
Nfkb1	1,2255	0,375081	0,293370484
Nfkb2	2,1189	0,049813	1,083315502
Nfkbia	3,0738	0,042033	1,620023298
Nfkbib	1,8277	0,073125	0,870029285
Nlrc5	1,0968	0,537277	0,133300476
Nlrp12	0,8685	0,521313	-0,203402246
Otud7b	1,154	0,398019	0,206643224
Rel	0,7544	0,419906	-0,406598419
Relb	1,8361	0,088699	0,876644635
Snf8	0,9159	0,839811	-0,126738005
Sp1	0,8141	0,574751	-0,296722076
Sp110	2,5847	0,049534	1,36999684
Stap2	2,6882	0,09493	1,426640478
Stat1	1,398	0,27485	0,483364361
Stat6	1,257	0,380284	0,32998465
Syk	1,7371	0,123443	0,796680808
Tbk1	1,2658	0,309278	0,340049473
Tlr4	0,7937	0,445875	-0,333334289
Tmem173	0,5743	0,135181	-0,800123533
Tnfrsf11a	0,79	0,233864	-0,340075442
Tnfrsf12a	1,4406	0,26372	0,526669809
Tnfrsf13c	0,8685	0,521313	-0,203402246
Traf1	4,7678	0,020252	2,253323719
Traf2	1,1173	0,588151	0,160016608
Traf3	0,893	0,712742	-0,16326792
Traf6	1,021	0,842702	0,029982866
Vegfb	0,9202	0,767881	-0,119980638
Xbp1	0,7153	0,233946	-0,483379653
Yy1	0,9727	0,95349	-0,039933177
Rn18s	1	0	

The expression of the analyzed genes was evaluated using RT-qPCR. The cells were left untreated or treated with poly(I:C) for 18 h. Significant changes ($p \leq 0.05$, $\log_2$ fold change ≥ 1) in gene expression are shown in red (upregulation). *Rn18s* – reference gene. Data were obtained from three independent experiments.

Supplementary Table 2. Expression of genes involved in NF-κB signaling pathways in RAW 264.7 macrophages stimulated with IFN+LPS for 18 h.

IFN+LPS vs control			
**Gene**	**Fold change**	***p* value**	**Log_2 fold change**
Aim2	1,1755	0,376043	0,233274539
Atf3	8,0556	0,001672	3,009992048
Bcl2	1,1947	0,443994	0,25664839
Bcl3	4,3671	0,002542	2,126675567
Birc2	1,1173	0,485158	0,160016608
Birc3	2,0046	0,022456	1,003314389
Ccl19	0,79	0,277318	-0,340075442

IFN+LPS vs control			
**Gene**	**Fold change**	***p* value**	**Log$_2$ fold change**
Ccl25	0,732	0,037062	-0,450084446
Ccr10	0,79	0,277318	-0,340075442
Ccr8	0,79	0,277318	-0,340075442
Cd36	0,7992	0,003956	-0,323371512
Cd40	14,1559	0,000531	3,82333157
Cd68	1,0792	0,669441	0,109962253
Cd86	24,0284	0,004954	4,586668681
Cflar	3,387	0,0008	1,760007987
Chuk	1,0867	0,625717	0,119953718
Creb1	1,0693	0,712791	0,096666669
Csf2	29,5824	0,023173	4,886667197
Cxcl10	10,3627	0,099441	3,373328041
Cxcl5	0,79	0,277318	-0,340075442
Cxcr5	0,5023	0,014451	-0,99337882
Daxx	3,3558	0,000113	1,746656736
Ddx58	13,7688	0,000444	3,783330924
Ets2	3,793	0,001497	1,923339372
Hdac9	0,0976	0,000009	-3,356975042
Hmgb1	0,2137	0,000341	-2,226341187
Icam1	0,4622	0,004463	-1,113410835
Ido1	0,79	0,277318	-0,340075442
Ifit1	321,7954	0,003255	8,329999893
Ifnb1	0,79	0,277318	-0,340075442
Ifngr1	0,3669	0,019232	-1,44654119
Ikbkb	0,8312	0,242412	-0,266732441
Ikbke	2,362	0,006043	1,240008965
Ikbkg	3,2944	0,01148	1,720015735
Il15	3,5884	0,008991	1,843340717
Il18	3,3404	0,017744	1,74002087
Il9	0,79	0,277318	-0,340075442
Irak1	0,52	0,018042	-0,943416472
Irak3	8,187	0,00222	3,033334895
Irf1	1,2142	0,029227	0,280006078
Irf2	0,5535	0,035182	-0,853344778
Irf3	0,7614	0,108418	-0,393273525
Irf5	0,4137	0,00059	-1,273343137
Irf8	1,9908	0,004015	0,993348292
Jak1	2,1987	0,002813	1,13665077
Ltbr	1,0892	0,664786	0,123268887
Map2k3	0,3807	0,005289	-1,393273525
Mapkapk2	1,3787	0,045093	0,463308566
Mavs	1,2894	0,27235	0,366699889
Mmd2	0,79	0,277318	-0,340075442
Mmp2	0,79	0,277318	-0,340075442
Mmp25	4,2673	0,010628	2,093323539
Mmp9	71,3414	0,005554	6,156667622
Mtor	0,7055	0,147103	-0,503282012
Myd88	0,7087	0,10163	-0,496753046
Nfat5	1,3134	0,011848	0,39330636
Nfix	0,79	0,277318	-0,340075442
Nfkb1	2,0753	0,011231	1,053319904
Nfkb2	4,3974	0,003583	2,13665077
Nfkbia	9,2964	0,000836	3,216672146

IFN+LPS vs control			
Gene	Fold change	*p* value	Log_2 fold change
Nfkbib	4,7459	0,000054	2,246681702
Nlrc5	3,6217	0,000655	1,856667047
Nlrp12	0,79	0,277318	-0,340075442
Otud7b	1,617	0,016423	0,693319679
Rel	2,0326	0,015182	1,023326332
Relb	1,4641	0,04962	0,550014095
Snf8	0,9033	0,197377	-0,146722886
Sp1	0,7509	0,151336	-0,413307303
Sp110	5,3517	0,000013	2,419997245
Stap2	2,8481	0,004973	1,509999802
Stat1	4,5842	0,001479	2,196669988
Stat6	1,0473	0,726421	0,066674763
Syk	8,0556	0,000924	3,009992048
Tbk1	2,114	0,002412	1,079975377
Tlr4	1,4175	0,051654	0,503348735
Tmem173	0,8179	0,402	-0,290003631
Tnfrsf11a	0,3703	0,000024	-1,433233545
Tnfrsf12a	0,8586	0,212509	-0,219941922
Tnfrsf13c	0,79	0,277318	-0,340075442
Traf1	9,736	0,000331	3,283329168
Traf2	1,2834	0,043059	0,359970888
Traf3	0,6537	0,051457	-0,613299398
Traf6	1,8025	0,001163	0,849999259
Vegfb	0,7614	0,21571	-0,393273525
Xbp1	0,6043	0,068131	-0,726663153
Yy1	0,7614	0,089189	-0,393273525
Rn18s	1	0	

The expression of the analyzed genes was evaluated using RT-qPCR. The cells were left untreated or treated with rmIFN-γ + *Escherichia coli* LPS O111:B4 for 18 h. Significant changes ($p \leq 0.05$, $\log_2$ fold change ≤ -1 or ≥ 1) in gene expression are shown in colors (blue-downregulation, red-upregulation). *Rn18s* – reference gene. Data were obtained from three independent experiments.

Supplementary Table 3. Expression of genes involved in NF-κB signaling pathways in RAW 264.7 macrophages infected with ECTV (18 hpi).

ECTV vs control			
Gene	Fold change	*p* value	Log_2 fold change
Aim2	1,1173	0,602205	0,160016608
Atf3	1,3755	0,29558	0,45995614
Bcl2	0,9482	0,770468	-0,076736702
Bcl3	0,8526	0,326947	-0,23005904
Birc2	0,8706	0,339986	-0,199918075
Birc3	0,4147	0,002874	-1,269860048
Ccl19	0,8217	0,336514	-0,283316328
Ccl25	1,2454	0,204151	0,316609184
Ccr10	0,8217	0,336514	-0,283316328
Ccr8	0,8217	0,336514	-0,283316328
Cd36	0,8274	0,534445	-0,273343137
Cd40	0,9615	0,786884	-0,056641237
Cd68	0,9772	0,975382	-0,033274231
Cd86	0,9461	0,788723	-0,079935415
Cflar	1,2114	0,482786	0,276675317
Chuk	0,8766	0,541925	-0,190009416

ECTV vs control			
**Gene**	**Fold change**	***p* value**	**Log_2 fold change**
Creb1	0,8467	0,357689	-0,240077206
Csf2	0,8217	0,336514	-0,283316328
Cxcl10	0,4147	0,010591	-1,269860048
Cxcl5	0,8217	0,336514	-0,283316328
Cxcr5	0,8123	0,149095	-0,299915451
Daxx	0,9054	0,650766	-0,143372788
Ddx58	1,0644	0,958966	0,090040415
Ets2	0,8546	0,209773	-0,226678778
Hdac9	0,7974	0,34211	-0,32662449
Hmgb1	1,0305	0,789881	0,043344505
Icam1	0,5824	0,008824	-0,779917739
Ido1	0,8217	0,336514	-0,283316328
Ifit1	1,1755	0,531604	0,233274539
Ifnb1	0,8217	0,336514	-0,283316328
Ifngr1	0,9862	0,901123	-0,020047842
Ikbkb	0,8971	0,566751	-0,156659283
Ikbke	0,4527	0,010334	-1,143372788
Ikbkg	0,4965	0,008475	-1,010134377
Il15	1,0093	0,953586	0,013355059
Il18	0,8625	0,647426	-0,213403638
Il9	0,8217	0,336514	-0,283316328
Irak1	0,9593	0,88127	-0,059946038
Irak3	0,839	0,206032	-0,253257284
Irf1	0,8606	0,193443	-0,216585255
Irf2	0,8766	0,396661	-0,190009416
Irf3	0,9181	0,617204	-0,123276793
Irf5	0,7614	0,178038	-0,393273525
Irf8	1,2716	0,328404	0,346644922
Jak1	1,0994	0,646682	0,136716384
Ltbr	1,1277	0,608658	0,173383321
Map2k3	0,9593	0,770533	-0,059946038
Mapkapk2	1,0163	0,920971	0,023326332
Mavs	1,0521	0,89971	0,073271836
Mmd2	0,8217	0,336514	-0,283316328
Mmp2	0,8217	0,336514	-0,283316328
Mmp25	0,8217	0,336514	-0,283316328
Mmp9	0,8217	0,336514	-0,283316328
Mtor	1,0449	0,923134	0,063364879
Myd88	0,9395	0,768999	-0,090034933
Nfat5	0,9374	0,532508	-0,0932633
Nfix	0,8217	0,336514	-0,283316328
Nfkb1	0,8507	0,597675	-0,233277641
Nfkb2	0,395	0,01653	-1,340075442
Nfkbia	0,4434	0,005844	-1,173319325
Nfkbib	0,6814	0,026913	-0,553426147
Nlrc5	0,9548	0,921309	-0,066729528
Nlrp12	0,8217	0,336514	-0,283316328
Otud7b	0,9461	0,743349	-0,079935415
Rel	1,0281	0,895858	0,039980598
Relb	0,5129	0,012834	-0,963250524
Snf8	0,9309	0,930681	-0,103301897
Sp1	1,0305	0,858416	0,043344505
Sp110	0,8448	0,437815	-0,24331826

ECTV vs control			
**Gene**	**Fold change**	***p* value**	**Log2 fold change**
Stap2	0,7774	0,373436	-0,363270987
Stat1	0,9117	0,541575	-0,133368919
Stat6	0,9615	0,858482	-0,056641237
Syk	1,3787	0,209777	0,463308566
Tbk1	0,9931	0,975308	-0,009989098
Tlr4	0,8428	0,304096	-0,246737781
Tmem173	1,1783	0,51165	0,236706902
Tnfrsf11a	0,5	0,008603	-1
Tnfrsf12a	0,5743	0,048308	-0,800123533
Tnfrsf13c	0,8217	0,336514	-0,283316328
Traf1	0,2031	0,001663	-2,299737855
Traf2	0,7337	0,262713	-0,44673781
Traf3	0,9013	0,484798	-0,149920704
Traf6	1,1728	0,275573	0,229957009
Vegfb	1,2628	0,382737	0,336626166
Xbp1	0,8274	0,366386	-0,273343137
Yy1	0,9885	0,990923	-0,016687129
Rn18s	1	0	

The expression of the analyzed genes was evaluated using RT-qPCR. The cells were uninfected or infected with ECTV for 18 h. Significant changes ($p \leq 0.05$, $\log_2$ fold change ≤ -1) in gene expression are shown in colors (blue-downregulation). *Rn18s* – reference gene. Data were obtained from three independent experiments.

Supplementary Table 4. Expression of genes involved in NF-κB signaling pathways in RAW 264.7 macrophages infected with ECTV and stimulated with poly(I:C) (18 hpi).

ECTV/Poly(I:C) vs Poly(I:C)			
**Gene**	**Fold change**	***p* value**	**Log2 fold change**
Aim2	0,6285	0,212908	-0,67001535
Atf3	0,4774	0,022207	-1,066729528
Bcl2	0,5023	0,172089	-0,99337882
Bcl3	0,7405	0,301668	-0,433428359
Birc2	0,4434	0,074796	-1,173319325
Birc3	0,2343	0,030834	-2,093571141
Ccl19	0,9977	0,912319	-0,00332202
Ccl25	0,8332	0,585547	-0,263265256
Ccr10	0,9977	0,912319	-0,00332202
Ccr8	0,9977	0,912319	-0,00332202
Cd36	0,8236	0,381239	-0,279984265
Cd40	0,2387	0,035577	-2,066729528
Cd68	0,4393	0,084845	-1,186721595
Cd86	0,9977	0,912319	-0,00332202
Cflar	0,5012	0,091022	-0,99654168
Chuk	0,4665	0,048302	-1,100051014
Creb1	0,5212	0,069633	-0,940091011
Csf2	0,9977	0,912319	-0,00332202
Cxcl10	0,2535	0,062131	-1,979942348
Cxcl5	0,9977	0,912319	-0,00332202
Cxcr5	0,6926	0,549072	-0,529905707
Daxx	0,4147	0,061215	-1,269860048
Ddx58	0,1032	0,038097	-3,276485124
Ets2	0,483	0,047249	-1,049904906
Hdac9	0,5421	0,056488	-0,883369088

ECTV/Poly(I:C) vs Poly(I:C)			
**Gene**	**Fold change**	***p* value**	**Log_2 fold change**
Hmgb1	0,8971	0,572062	-0,156659283
Icam1	0,3914	0,034135	-1,353284339
Ido1	0,9977	0,912319	-0,00332202
Ifit1	0,08	0,060512	-3,64385619
Ifnb1	0,9977	0,912319	-0,00332202
Ifngr1	0,4931	0,104808	-1,020047842
Ikbkb	0,4137	0,05596	-1,273343137
Ikbke	0,2553	0,034131	-1,969734557
Ikbkg	0,3157	0,008588	-1,663373834
Il15	0,1715	0,018517	-2,543719518
Il18	0,2606	0,165576	-1,940091011
Il9	0,9977	0,912319	-0,00332202
Irak1	0,531	0,138692	-0,913216234
Irak3	0,5664	0,156333	-0,820106829
Irf1	0,52	0,146167	-0,943416472
Irf2	0,5421	0,120486	-0,883369088
Irf3	0,5285	0,122142	-0,920024623
Irf5	0,5212	0,092894	-0,940091011
Irf8	0,5797	0,142175	-0,786621609
Jak1	0,4988	0,23652	-1,00346663
Ltbr	0,6492	0,190703	-0,623265095
Map2k3	0,5371	0,080589	-0,896737373
Mapkapk2	0,4313	0,038549	-1,213236379
Mavs	0,5535	0,138558	-0,853344778
Mmd2	0,9977	0,912319	-0,00332202
Mmp2	0,9977	0,912319	-0,00332202
Mmp25	0,9977	0,912319	-0,00332202
Mmp9	0,0387	0,002466	-4,691522623
Mtor	0,5285	0,14745	-0,920024623
Myd88	0,5082	0,075377	-0,976531719
Nfat5	0,5757	0,186766	-0,796610883
Nfix	0,9977	0,912319	-0,00332202
Nfkb1	0,4475	0,058497	-1,160040413
Nfkb2	0,2558	0,014396	-1,966911831
Nfkbia	0,2398	0,030497	-2,060096436
Nfkbib	0,3415	0,027322	-1,550042516
Nlrc5	0,5837	0,055837	-0,776701027
Nlrp12	0,9977	0,912319	-0,00332202
Otud7b	0,6142	0,061027	-0,703219583
Rel	0,8179	0,45206	-0,290003631
Relb	0,4774	0,065263	-1,066729528
Snf8	0,577	0,112781	-0,793356776
Sp1	0,6085	0,174744	-0,716670832
Sp110	0,2248	0,022922	-2,153286059
Stap2	0,1466	0,033616	-2,770042991
Stat1	0,2845	0,038976	-1,813499442
Stat6	0,473	0,075463	-1,080087911
Syk	0,3439	0,026521	-1,539938979
Tbk1	0,4852	0,048226	-1,043348544
Tlr4	0,5561	0,111615	-0,846583758
Tmem173	0,6358	0,247556	-0,653355078
Tnfrsf11a	0,5023	0,038571	-0,99337882
Tnfrsf12a	0,4323	0,090839	-1,209895259

ECTV/Poly(I:C) vs Poly(I:C)			
**Gene**	**Fold change**	***p* value**	**Log2 fold change**
Tnfrsf13c	0,9977	0,912319	-0,00332202
Traf1	0,2181	0,022437	-2,196938325
Traf2	0,4414	0,066839	-1,179841465
Traf3	0,7423	0,252861	-0,429925726
Traf6	0,7137	0,206748	-0,486610322
Vegfb	0,5046	0,064417	-0,986787889
Xbp1	0,4676	0,048256	-1,096653165
Yy1	0,6752	0,206195	-0,566613191
Rn18s	1	0	

The expression of the analyzed genes was evaluated using RT-qPCR. The cells were uninfected and or infected with ECTV and were left untreated or treated with poly(I:C) for 18 h. Significant changes ($p \leq 0.05$, $\log_2$ fold change ≤ -1) in gene expression are shown in blue (downregulation). *Rn18s* – reference gene. Data were obtained from three independent experiments.

Supplementary Table 5. Expression of genes involved in NF-κB signaling pathways in RAW 264.7 macrophages infected with ECTV and stimulated with IFN+LPS (18 hpi).

ECTV/IFN+LPS vs IFN+LPS			
**Gene**	**Fold change**	***p* value**	**Log2 fold change**
Aim2	0,5409	0,017001	-0,886566197
Atf3	0,4741	0,010668	-1,076736702
Bcl2	0,2365	0,001898	-2,080087911
Bcl3	0,6227	0,037635	-0,683390816
Birc2	0,3618	0,006656	-1,466735687
Birc3	0,1839	0,004017	-2,443006615
Ccl19	1,0234	0,81205	0,033370138
Ccl25	0,6057	0,001724	-0,723324683
Ccr10	1,0234	0,81205	0,033370138
Ccr8	1,0234	0,81205	0,033370138
Cd36	0,1856	0,000045	-2,429731384
Cd40	0,1975	0,000941	-2,340075442
Cd68	0,1684	0,001443	-2,570035956
Cd86	0,1672	0,008808	-2,580353247
Cflar	0,4024	0,000294	-1,31329779
Chuk	0,3172	0,004748	-1,656535324
Creb1	0,2117	0,004114	-2,239906826
Csf2	0,0504	0,024532	-4,310432456
Cxcl10	0,0167	0,083843	-5,904008087
Cxcl5	1,0234	0,81205	0,033370138
Cxcr5	1,4473	0,191448	0,533363998
Daxx	0,1715	0,000065	-2,543719518
Ddx58	0,0357	0,000326	-4,807932116
Ets2	0,483	0,010502	-1,049904906
Hdac9	0,722	0,067664	-0,469929258
Hmgb1	0,4538	0,005938	-1,139871486
Icam1	1,4044	0,174043	0,489953901
Ido1	1,0234	0,81205	0,033370138
SIfit1	0,0164	0,003422	-5,930160375
Ifnb1	1,0234	0,81205	0,033370138
Ifngr1	0,3959	0,000768	-1,336792028
Ikbkb	0,386	0,002503	-1,373327247
Ikbke	0,1966	0,001655	-2,346664773
Ikbkg	0,1627	0,006376	-2,619713844

ECTV/IFN+LPS vs IFN+LPS			
**Gene**	**Fold change**	***p* value**	**Log₂ fold change**
Il15	0,0994	0,003178	-3,330610338
Il18	0,1101	0,006546	-3,183113626
Il9	1,0234	0,81205	0,033370138
Irak1	0,3471	0,000436	-1,52657673
Irak3	0,1822	0,002975	-2,456405136
Irf1	0,2987	0,000473	-1,743230857
Irf2	0,2264	0,002904	-2,143054137
Irf3	0,2269	0,000139	-2,139871486
Irf5	0,3172	0,001794	-1,656535324
Irf8	0,1634	0,000034	-2,613520111
Jak1	0,2859	0,000175	-1,806417475
Ltbr	0,408	0,010363	-1,293358943
Map2k3	0,5522	0,161258	-0,856737207
Mapkapk2	0,2636	0,000016	-1,923577725
Mavs	0,25	0,000927	-2
Mmd2	1,0234	0,81205	0,033370138
Mmp2	1,0234	0,81205	0,033370138
Mmp25	0,2012	0,008294	-2,31329779
Mmp9	0,3353	0,024132	-1,576475612
Mtor	0,5129	0,003578	-0,963250524
Myd88	0,4434	0,009862	-1,173319325
Nfat5	0,2655	0,000185	-1,913216234
Nfix	1,0234	0,81205	0,033370138
Nfkb1	0,3754	0,006904	-1,413499445
Nfkb2	0,1525	0,002281	-2,713118852
Nfkbia	0,0838	0,000774	-3,576905946
Nfkbib	0,1917	0,000055	-2,383077758
Nlrc5	0,15	0,000383	-2,736965594
Nlrp12	1,0234	0,81205	0,033370138
Otud7b	0,7153	0,15712	-0,483379653
Rel	0,3643	0,008452	-1,4568011
Relb	0,6522	0,129553	-0,616613654
Snf8	0,3345	0,019971	-1,579921884
Sp1	0,3987	0,001931	-1,32662449
Sp110	0,1568	0,000013	-2,673002535
Stap2	0,1116	0,000085	-3,163591068
Stat1	0,0273	0,000541	-5,194955239
Stat6	0,3322	0,000669	-1,589876022
Syk	0,198	0,001391	-2,336427665
Tbk1	0,3106	0,000991	-1,686870265
Tlr4	0,2349	0,00018	-2,089881382
Tmem173	0,4414	0,024423	-1,179841465
Tnfrsf11a	0,1805	0,00039	-2,469929258
Tnfrsf12a	0,6001	0,040125	-0,736725165
Tnfrsf13c	1,0234	0,81205	0,033370138
Traf1	0,0921	0,000318	-3,440655033
Traf2	0,2932	0,000177	-1,770042991
Traf3	0,5141	0,006526	-0,959879083
Traf6	0,5	0,001708	-1
Vegfb	0,3577	0,008124	-1,483177977
Xbp1	0,1642	0,001041	-2,606473968
Yy1	0,6814	0,087199	-0,553426147
Rn18s	1	0	

The expression of the analyzed genes was evaluated using RT-qPCR. The cells were uninfected or infected with ECTV and were left untreated or treated with rmIFN-γ + *Escherichia coli* LPS O111:B4 for 18 h. Significant changes ($p \leq 0.05$, $\log_2$ fold change ≤ -1) in gene expression are shown in blue (downregulation). *Rn18s* – reference gene. Data were obtained from three independent experiments.
